# Alzheimer’s Patient Microglia Exhibit Enhanced Aging and Unique Transcriptional Activation

**DOI:** 10.1016/j.celrep.2020.107843

**Published:** 2020-06-30

**Authors:** Karpagam Srinivasan, Brad A. Friedman, Ainhoa Etxeberria, Melanie A. Huntley, Marcel P. van der Brug, Oded Foreman, Jonathan S. Paw, Zora Modrusan, Thomas G. Beach, Geidy E. Serrano, David V. Hansen

**Affiliations:** 1 Department of Neuroscience, Genentech, Inc., South San Francisco, CA, USA; 2 Department of Bioinformatics and Computational Biology, Genentech, Inc., South San Francisco, CA, USA; 3 Department of Biomarker Discovery OMNI, Genentech, Inc., South San Francisco, CA, USA; 4 Department of Pathology, Genentech, Inc., South San Francisco, CA, USA; 5 Department of Immunology, Genentech, Inc., South San Francisco, CA, USA; 6 Department of Microchemistry, Proteomics and Lipidomics, Genentech, Inc., South San Francisco, CA, USA; 7 Banner Sun Health Research Institute, Sun City, AZ, USA; 8 Present address: Alector, Inc., South San Francisco, CA, USA; 9 Present address: Clover Health, San Francisco, CA, USA; 10 Present address: Calico Life Sciences LLC, South San Francisco, CA, USA; 11 Present address: Brigham Young University, Provo, UT, USA; 12 These authors contributed equally; 13 Lead Contact

## Abstract

Damage-associated microglia (DAM) profiles observed in Alzheimer’s disease (AD)-related mouse models reflect an activation state that could modulate AD risk or progression. To learn whether human AD microglia (HAM) display a similar profile, we develop a method for purifying cell types from frozen cerebrocortical tissues for RNA-seq analysis, allowing better transcriptome coverage than typical single-nucleus RNA-seq approaches. The HAM profile we observe bears little resemblance to the DAM profile. Instead, HAM display an enhanced human aging profile, in addition to other disease-related changes such as *APOE* upregulation. Analyses of whole-tissue RNA-seq and single-cell/nucleus RNA-seq datasets corroborate our findings and suggest that the lack of DAM response in human microglia occurs specifically in AD tissues, not other neurodegenerative settings. These results, which can be browsed at http://research-pub.gene.com/BrainMyeloidLandscape, provide a genome-wide picture of microglial activation in human AD and highlight considerable differences between mouse models and human disease.

## In Brief

Gene expression analysis of human AD microglia has been limited by technical challenges. Srinivasan et al. use RNA-seq to profile FACS-purified microglia from frozen AD and control brains, revealing that human AD microglia exhibit accelerated aging, as well as age-independent changes, like upregulation of *APOE*.

## Graphical Abstract

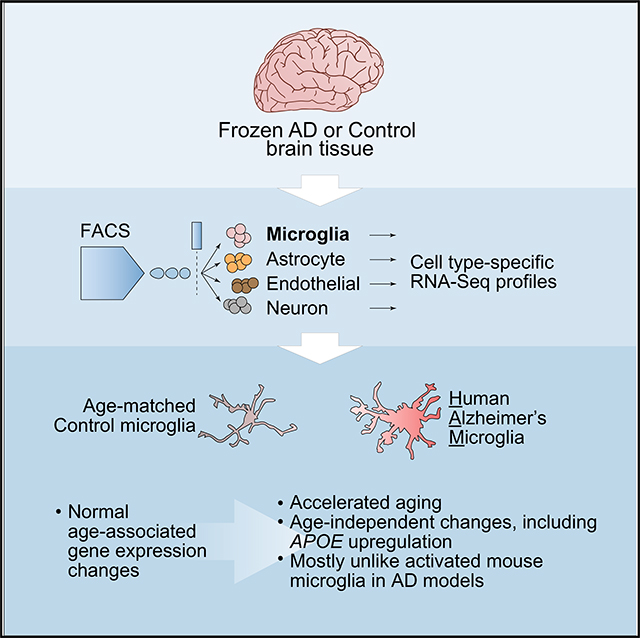

## INTRODUCTION

Human genetic studies have identified microglia, the brain’s resident myeloid cells, as a key cell type governing the risk of Alzheimer’s disease (AD) (Bellenguez et al., 2020;Hansen et al., 2018). Gene expression profiles in microglia from mouse models of AD are highly characterized and reflect specific myeloid cell activation states that could modulate AD risk or progression (Deczkowska et al., 2018; Friedman et al., 2018; Holtman et al., 2015; Keren-Shaul et al., 2017; Krasemann et al., 2017; Orre et al., 2014; Srinivasan et al., 2016; Wang et al., 2015). Although some groups have produced expression profiles for microglia from human brain tissues (Del-Aguila et al., 2019; Galatro et al., 2017; Gosselin et al., 2017; Jä kel et al., 2019; Masuda et al., 2019; Mathys et al., 2019; Olah et al., 2018; Zhang et al., 2016), the clarity with which we view microglial transcriptional states in mouse models of AD has not yet been realized for human AD tissues because of limited availability of fresh tissue samples and/or technological hurdles in recovering genome-wide transcriptomic data with cell-type resolution from frozen samples.

Here we employ a method for isolating multiple cell types from frozen, post-mortem human brain tissues, with the goal of profiling gene expression in microglia and other cell types from AD versus control tissues using RNA sequencing (RNA-seq). The method we developed allows the collection of desired cell types by the tens (or hundreds) of thousands from each tissue sample, provides rich gene expression profiles to enable genome-wide analyses of differential expression (DE), and allows the selection of sample cohorts that include a suitably large number of AD and control subjects with desired histopathological or clinical characteristics. A notable caveat of our method is the low quality of the RNA after its purification from the collected cell types because of unavoidable aspects of preparing fixed cell suspensions from frozen and thawed post-mortem tissue samples. Despite this caveat, we succeeded in using frozen specimens of human frontal cortex to characterize a human Alzheimer’s microglia (HAM) profile, which bore almost no resemblance to the damage-associated microglia (DAM) profile defined in mouse AD models. We validated our overall findings by qRT-PCR using separate preparations of microglia sorted from temporal cortex and using whole-tissue RNA-seq datasets from both frontal and temporal cortices. Extensive comparisons with other microglial RNA-seq datasets revealed that a distinct component of the HAM profile reflected an enhanced human aging phenotype. Finally, comparisons with recently published human microglia single-cell RNA sequencing (scRNA-seq) or single-nucleus RNA sequencing (snRNA-seq) datasets suggested that DAM gene induction was more evident in two other neurodegenerative settings. Thus, the relative lack of DAM gene induction in human microglia was a peculiar feature of human AD tissues and may be a specific feature of AD pathogenesis.

## RESULTS

### Defining the HAM Gene Expression Profile Using Frozen Tissues

We began with frozen samples of frontal cortex, which is affected by tau pathology in the later stages of disease (Braak stages V and VI) that roughly coincide with onset and progression of dementia. Tissue samples were excised from the superior frontal gyrus (SFG), which has been linked with visuospatial cognition both in AD and in lesion studies (du Boisgueheneuc et al., 2006; Valdé s Herná ndez et al., 2018). To maximize the likelihood of observing differences between AD and control, we selected only AD specimens with high scores for amyloid and tau neuropathology in frontal cortex, and we selected only control specimens with negligible amounts of these pathologies in this region (see sample metadata in Data S2). AD and control groups had roughly matching distributions of age, sex, and post-mortem interval (PMI).

**Figure 1.**
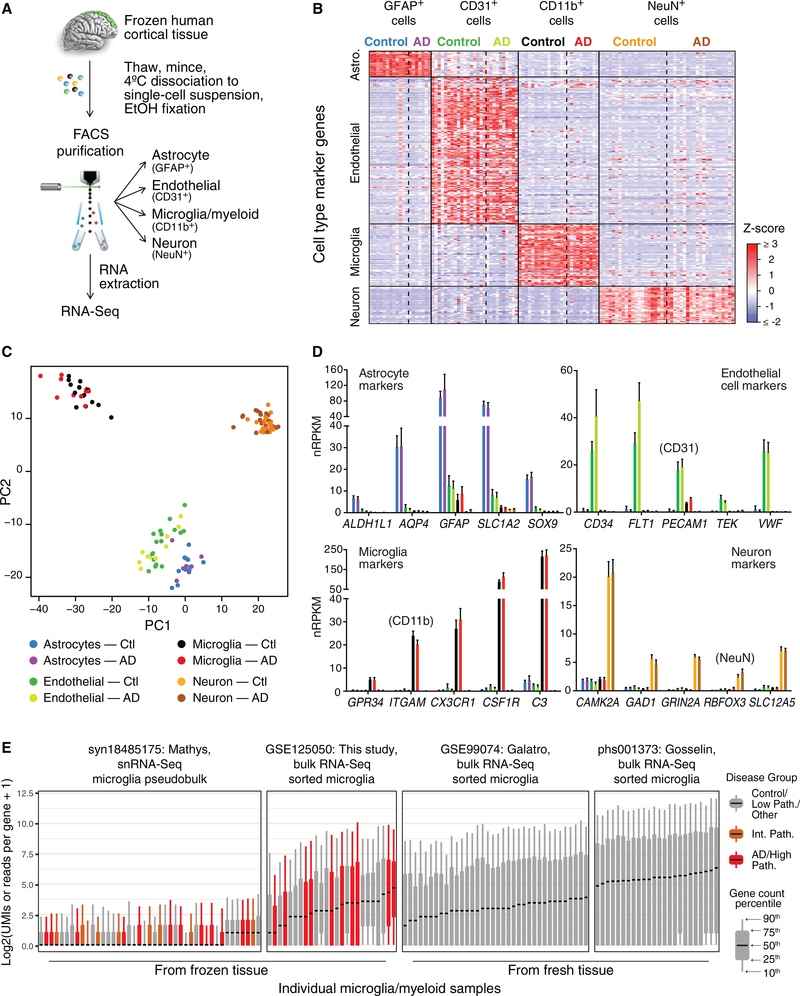
Expression Profiling of Human Cell Populations Sorted from Frozen, Post-Mortem SFG (A) Experimental overview. See Figure S1 for the FACS gating scheme. (B) Expression of known cell-type markers, derived from previously published human cell data from fresh brains (Zhang et al., 2016), in QC-passing expression profiles indicates high cell-type purity. Each gene was *Z* score normalized across all profiles of all cell types. See Figure S2 for QC analyses. (C) Principal-component analysis using most variable genes reveals separation of four cell types. The juxtaposition of astrocyte and endothelial cell profiles, and the modest detection of astrocyte markers in endothelial cell samples (see D), may have resulted from astrocytic endfeet (which contain mRNAs; Boulay et al., 2017) remaining associated with endothelial cell bodies. (D) Expression levels ± SEM of selected cell-type markers. (E) Distributions of gene counts in various human microglial gene expression datasets. Each boxplot shows the indicated (10th,25th,50th,75th, and 90th) quantile across all genes, of raw gene counts for each sample of bulk-sorted microglia or, for syn18485175, for each sample’s pseudobulk microglia.

For dissociating and sorting cell types from frozen human brain tissues, we adapted a method that we reported for fresh mouse brains involving brief enzymatic treatment at 4°C, mechanical dissociation, fixation in 50% ethanol, immunolabeling, and fluorescence-activated cell sorting (FACS) followed by RNA purification and sequencing (Srinivasan et al., 2016). Although the fixation adversely affects RNA integrity (RIN), it also permeabilizes the cells and enables labeling of intracellular markers for sorting. Labeling nuclei with DAPI helps ensure that only singlet cell bodies are collected, because doublets with a higher DAPI signal and cell fragments that lack nuclei are easily excluded. Using this method, we established a FACS gating strategy for collecting NeuN^+^ neurons, GFAP^+^ astrocytes, CD31^+^ endothelial cells, and CD11b^+^ microglia/myeloid cells (hereafter referred to simply as microglia) from thawed, dissociated SFG specimens (Figures 1A and S1). qRT-PCR for specific cell-type markers validated the specificity of the collected populations and was successful for nearly all RNA samples (data not shown). However, the RIN of the collected populations was poor, with Bioanalyzer RIN scores in the range of 1–3. Attempts to obtain better-quality RNA samples using alternative conditions for tissue digestion and cell fixation (see STAR Methods) were unsuccessful and usually counterproductive. We presume that damage from the freeze-thaw process led to cellular disintegration when cells remained unfixed for too long or were incubated at 37°C. Only brief dissociation at 4°C and fixation immediately thereafter permitted cell populations to suitably endure the subsequent immunolabeling and FACS procedures. We completed this process for 22 AD and 21 control SFG tissues.

We next prepared cDNA libraries using a kit with random primers, because the RNA was highly fragmented. After preparing and sequencing the libraries, we examined the RNA-seq data to determine whether our method had generated usable expression profiles. Although several unacceptable RNA profiles had to be discarded (see Figure S2, Data S1, and STAR Methods), we obtained 113 cell-type-specific expression profiles, including microglia cell profiles from 15 control and 10 AD subjects (Figures 1B and 1C; Data S2). Cell-type-specific marker expression suggested that the RNA-seq profiles we retained represented the intended cell populations with reasonable fidelity (Figure 1D). Comparisons with recently published datasets indicated that our bulk-sorted microglia profiles from frozen tissues displayed coverage of the transcriptome similar to that of bulk-sorted microglia from fresh post-mortem tissues (Galatro et al., 2017; Gosselin et al., 2017) and better coverage than snRNA-seq profiles (combining all microglial nuclei from a given patient into a pseudobulk profile) obtained from frozen tissues (Mathys et al., 2019)(Figure 1E).

**Figure 2.**
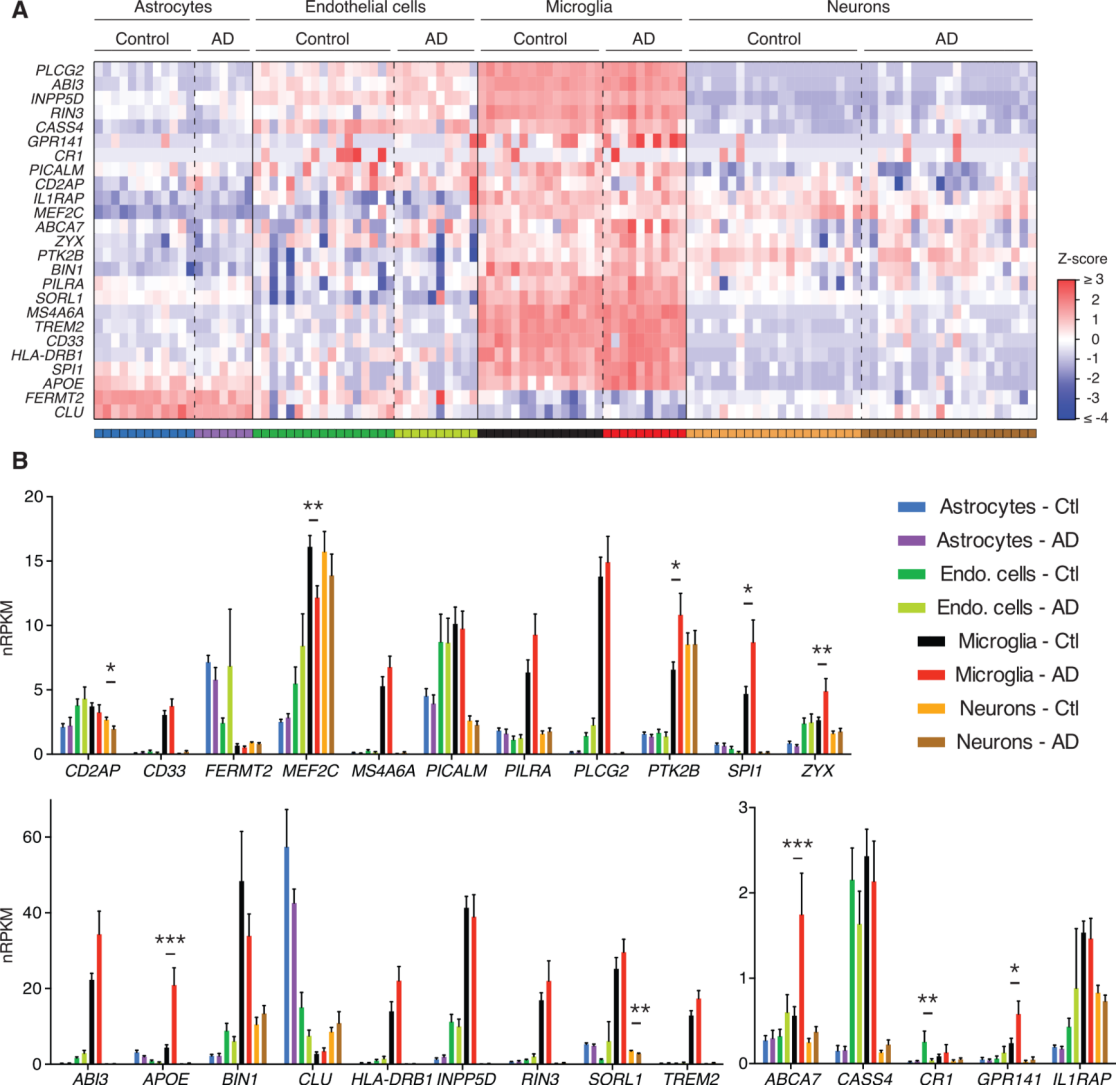
Sorted Cells from Frozen Human SFG Specimens Exhibit Preferential Expression of Many AD Risk Genes in Microglia (A) Heatmap of *Z* scores for each AD risk gene’s normalized reads per kilobase gene model per million total reads (nRPKM) expression value in each sample, with a sample’s *Z* score for a given gene representing its distance in standard deviations from the mean expression value across all samples for that gene. Gene selection was informed by genome-wide association study (GWAS) reports (Hollingworth et al., 2011; Lambert et al., 2013; Naj et al., 2011; Ramanan et al., 2015; Sims et al., 2017) and specific efforts to identify causal genes in GWAS-identified loci ( Huanget al., 2017; Novikova et al., 2019; Rathore et al., 2018). (B) Expression values are plotted for each AD risk gene in each cell type sorted from frozen SFG of controls (Ctl) or AD patients. Bars and lines represent mean expression ± SEM, with asterisks marking DE in AD versus control cells based on unadjusted DESeq2 p values (*p < 0.05, **p < 0.01, ***p < 0.001).

We examined the expression of twenty-five genes known or postulated to be associated with AD risk or progression (Hollingworth et al., 2011; Huang et al., 2017; Lambert et al., 2013; Naj et al., 2011; Novikova et al., 2019; Ramanan et al., 2015; Rathore et al., 2018; Sims et al., 2017). Similar to our analysis of a published human RNA-seq dataset that profiled cell types purified from freshly resected brain tissue (Hansen et al., 2018; Zhang et al., 2016), most AD risk genes in our cell types purified from frozen brain tissues showed preferential expression in microglia compared with other brain cell types (Figure 2A). We also examined whether any of these genes displayed altered expression levels in AD versus control cells, and we observed that *APOE*, *ABCA7*, *GPR141*, *PTK2B*, *SPI1*, and *ZYX* appeared upregulated in AD microglia, whereas *MEF2C* appeared downregulated (unadjusted p < 0.05) (Figure 2B). Using these criteria, we also observed downregulation of *CD2AP* and *SORL1* in AD neurons and of *CR1* in AD endothelial cells (Figure 2B).

**Figure 3.**
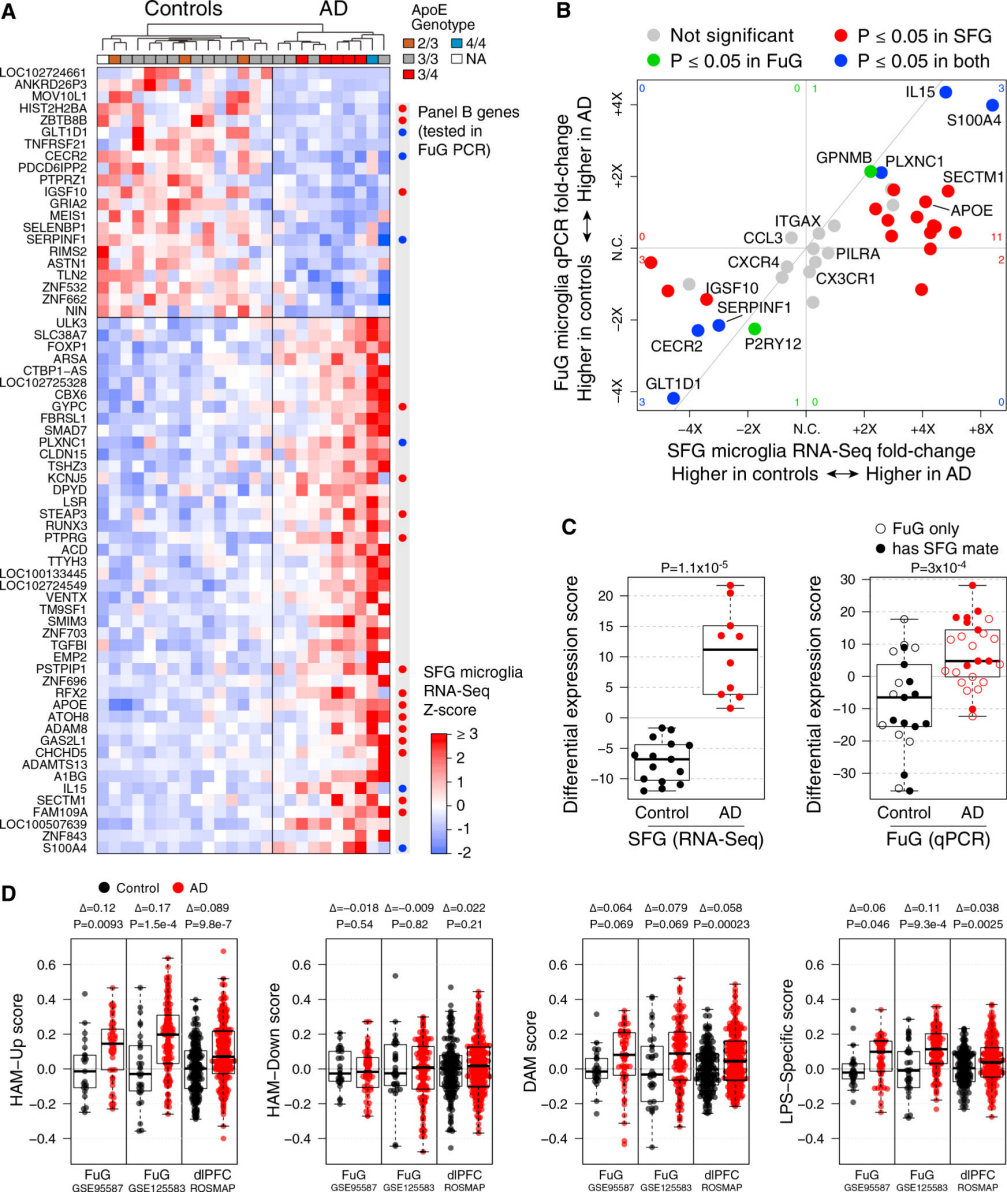
Human Microglia Exhibit an AD-Associated DE Profile in Both Frontal and Temporal Cortices (A) Heatmap of AD DE genes (rows; DESeq2 adjusted p ≤ 0.05 and maximum Cook’s p ≥ 0.01) in control and AD SFG-derived microglia expression profiles (columns, sorted by AD-associated DE). “Panel B genes” indicates genes that were subsequently assayed by qPCR in microglia sorted from FuG tissues, with colors from (B). (B) 4-way comparison of AD-associated DE in SFG microglia measured by RNA-seq (x axis) with DE in FuG microglia measured by qPCR (y axis). Each point represents one gene colored by whether the adjusted p value was ≤ 0.05 in one or both DE analyses (red for SFG RNA-seq, green for FuG qPCR, or blue for both). Corresponding numbers of DE genes are shown near the borders of the plot. For example, the red 11 on the right reflects the number of genes that were significantly up in SFG and trended up but did not meet significance in FuG, whereas the blue 3 at the top right indicates the number of genes significantly upin both regions. Genes were selected manually for validation, consisting of about 1/3 of the DE genes from the RNA-seq study and several other cell-type markers and genes of interest. Diagonal line: y = x. (See Figure S3A for subject-wise SFG-FuG microglia DE correlations, Figure S3B for selected qPCR data plots, and Data S2 columns EK–GH for qPCR expression statistics for all 39 genes in the panel.) (C) SFG microglia DE is reproduced in FuG microglia. DE scores (see STAR Methods) are shown for each SFG and FuG microglia sample, using the 22 SFG DE genes that were included in the qPCR panel. For FuG microglia samples, open circles indicate that a QC-passing SFG RNA-seq microglia profile was not available from that subject. p value, t test. (D) Detection of upregulated HAM profile genes is recapitulated in myeloid-balanced whole AD tissues from frontal and temporal cortical regions and is more robust than DAM changes predicted by mouse microglia profiles. Each study was separately myeloid balanced to create a subset of whole-tissue samples with similar myeloid gene set scores, and neuronal genes were removed from each gene set. (See Figure S4C for division by Braak stage with all samples and all genes included.) Each panel shows gene set scores for the indicated gene sets for each of the myeloid-balanced AD or control samples. Δ, mean log2 fold change; p value, t test.

Genome-wide analysis of DE using DESeq2 identified 45 genes increased and 21 genes decreased in AD microglia relative to controls (Figure 3A; for genome-wide expression values and DE statistics, see Data S2 and S3 for individual samples and group summaries, respectively). Of the changes in AD risk genes mentioned earlier, only *APOE* upregulation in microglia remained significant after correction for genome-wide testing (fold change = 4.1, adjusted p = 0.0004). We tested for contributions of age, sex, PMI, and *APOE* genotype to the DE profile, but none of these covariates accounted for the DE observed between AD and control groups (although a small number of other genes unrelated to AD status showed DE with age, sex, PMI, or *APOE* genotype) (see Data S4 and STAR Methods). We refer to the pattern of DE as the HAM profile.

### Validation of the HAM Profile in Multiple Cortical Regions and Datasets

We next tested whether the HAM profile detected in AD microglia from frontal cortex could be validated in microglia from temporal cortex, which is affected earlier in disease and may contain more downstream events by the time of death (Braak and Braak, 1991). Our temporal cortex samples were excised from the fusiform gyrus (FuG), which is important for object and face recognition (Chang et al., 2016), using many of the same subjects as the SFG tissues and totaling 25 AD and 21 control tissues. We generated another set of sorted-cell RNA samples using the method described earlier and performed qRT-PCR instead of RNA-seq to quantify transcript abundance for genes of interest, including a subset of DE genes from the SFG HAM profile (marked in Figure 3A). Despite the different disease contexts of FuG and SFG tissues, the direction of effect for AD versus control across RNA samples from CD11b^+^ cells was replicated for nearly every DE gene tested (Figures 3B and S3B). Using these 22 genes to assign a DE score to each sample revealed a clear difference in FuG microglia between AD and control groups, reproducing the signal observed in the SFG RNA-seq data (Figure 3C). Moreover, for subjects with both SFG RNA-seq and FuG PCR data available, the microglia DE scores were correlated between the two regions (Figure S3A). These findings alleviated potential concern about expression artifacts being introduced during RNA-seq library preparation.

A second way to validate the HAM profile was to examine whole-tissue RNA datasets from AD and control patients. Despite their limitations (Srinivasan et al., 2016), such datasets allow the evaluation of larger cohorts. We examined three studies: our previously published cohort of FuG samples (GEO: GSE95587), a newly generated FuG cohort (GEO: GSE125583) (see Figure S4A for reproducibility of whole-tissue DE profiles between FuG cohorts), and the Religious Orders Study and Rush Memory and Aging Project (ROSMAP) cohort (De Jager et al., 2018) from the dorsolateral prefrontal cortex. We used myeloid balancing (Friedman et al., 2018) to control for differences in myeloid cell abundance between control and AD tissues (Figure S4B), and exclusion of neuronal-enriched genes to mitigate the confounding effects of neuronal loss, before calculating gene set scores. In all three whole-tissue datasets, the HAM-Up gene set was significantly increased (Figure 3D); this was most apparent in later Braak stages, which also showed decreased expression of our HAM-Down gene set (Figure S4C). (The Braak stage analyses did not include the corrective measures for altered cellular makeup of AD tissues, because it was impractical to reduce the sample size.) These analyses provide additional evidence that our DE findings in AD microglia sorted from SFG and FuG tissues are not simply a peculiar feature of the small number of subjects (10 AD and 15 control) analyzed in our SFG RNA-seq dataset and that they do not result from biases in sampling of microglial subpopulations following tissue dissociation and FACS; they are instead real gene expression changes seen in both temporal and frontal cortical tissue.

Having detected the elevated HAM signature in AD whole-tissue RNA, we next wanted to see how its detection compared with that of known microglial expression modules recently identified in mouse models. We reported that expression signals for some of these modules, including the neurodegeneration-related (DAM) module and a lipopolysaccharide (LPS)-specific gene set, were slightly elevated in whole-tissue RNA profiles from AD brains (Friedman et al., 2018). We observed that the HAM-Up gene set was more robustly elevated in AD whole-tissue RNA than either of the mouse-derived gene sets (Figures 3D and S4C), underscoring the relevance of the DE results we observed in our sorted SFG microglia.

### The HAM Profile Is Unlike Known Mouse Microglial Activation States

**Figure 4.**
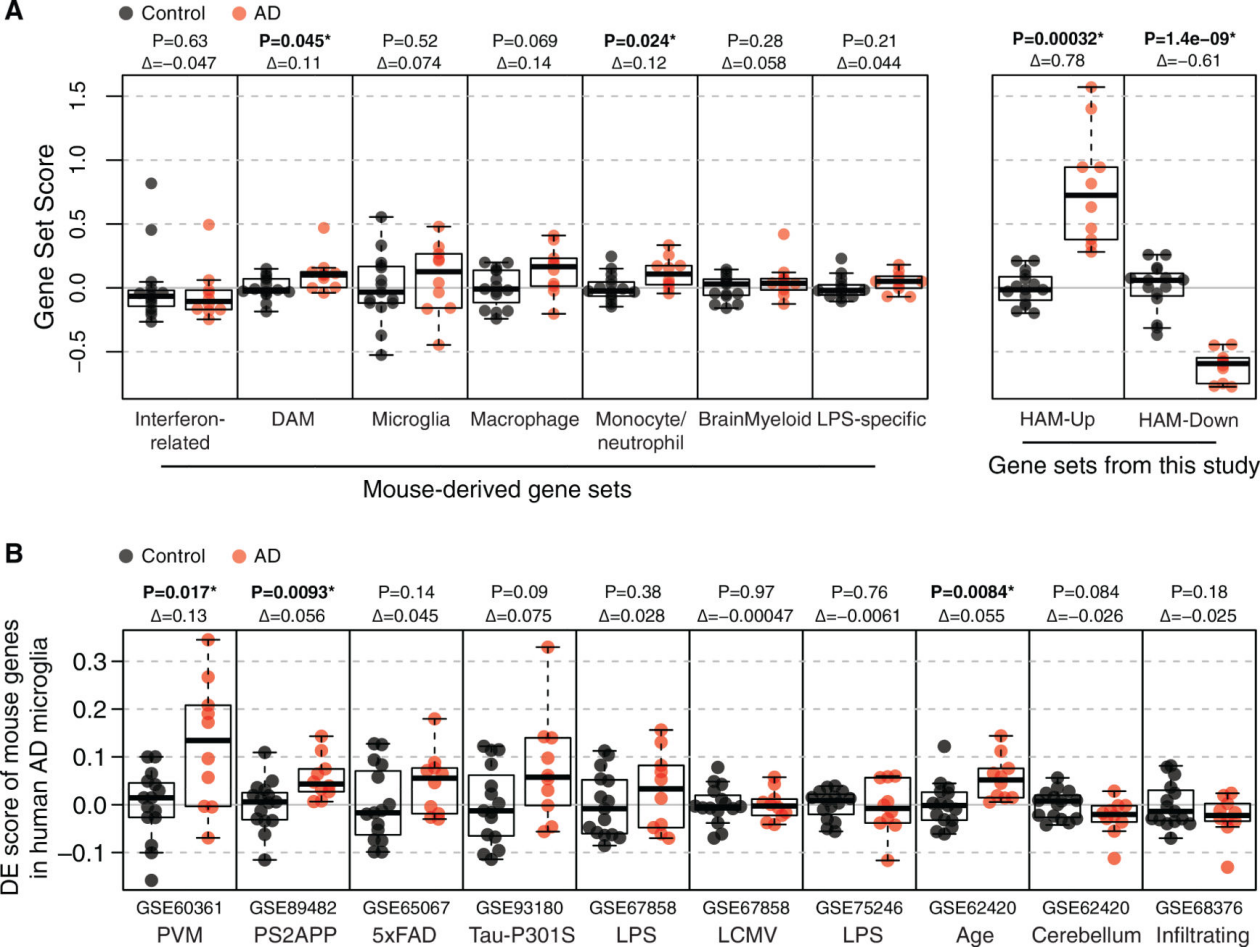
DE Genes in Mouse Microglia Studies Are Mostly Unchanged in HAM (A) Distribution of scores for mouse- and human-derived gene sets in SFG microglia profiles indicates that mouse-derived microglia gene modules undergo little or no change in AD microglia. The DAM gene set was called "neurodegeneration-related" in the previous manuscript. p, (unadjusted) t test; Δ, log2 fold changes in score; *p ≤ 0.05. (See Figures S5A–S5C for heatmaps of individual genes from DAM, microglia, and BrainMyeloid modules.) (B) DE gene set scores, similar to (A) but with DE genes from specific mouse datasets instead of from meta-analysis-derived gene modules. In this case, the scores are DE scores, meaning that they used signed means rather than means (with the sign indicating the direction of DE) so that up- and downregulated genes can be considered together. PVMs relative to parenchymal microglia; age, 22 months relative to young (≤12 month) microglia; cerebellum relative to cortical microglia; infiltrating macrophages (induced by irradiation) relative to tissue-resident microglia. p, (unadjusted) t test; Δ, log2 fold changes in score; *p ≤ 0.05. (For the three comparisons that reached significance, see Figure S5D for 4-way plots of individual gene fold changes in the respective mouse study compared to fold changes in AD versus control SFG microglia. See also Figure S6 for analysis of whether DE genes from the HAM profile are altered in mouse microglia in models of neurodegeneration or other activating conditions.)

We next used gene set score analysis to look for overlap in DE patterns between our AD versus control human microglia profiles and DE patterns observed in mouse microglia. First, we tested whether modulation of the mouse-derived gene modules we defined in a previous meta-analysis (Friedman et al., 2018) might be more apparent in our sorted microglia profiles than in the whole-tissue profiles described earlier, but any such AD-related changes were again subtle if present—especially compared with the HAM-Up and HAM-Down gene sets (Figure 4A). For example, expression of the mouse neurodegeneration-related/DAM module was slightly increased in AD microglia, just reaching significance (p = 0.045). However, of more than 100 genes in the module, only *APOE* was significantly increased in SFG microglia from AD patients (fold change = 4.1, p = 0.0004), and most other genes showed no clear trends in either direction (Figure S5A; Table S1). Similarly, although we observed a subtle increase in expression for the monocyte/ neutrophil module in AD microglia (Figure 4A), no individual genes in the module showed DE with genome-wide significance (Table S1). The microglia and brain myeloid gene modules that define resting or homeostatic microglia and are downregulated in response to virtually any perturbation in mice (Friedman et al., 2018) showed no hint of downregulation in AD microglia (Figure 4A). Of more than 150 genes in these modules, only *SERPINF1* (fold change = 0.35, p = 0.0062) showed the significant reduction predicted by mouse data (Figures S5B and S5C; Table S1).

We also cross-checked specific mouse studies for potential relationships between our HAM profiles and DE genes associated with PS2APP or 5xFAD β-amyloid model microglia, Tau-P301S frontotemporal dementia (FTD) model microglia, microglia following LPS or lymphocytic choriomeningitis virus (LCMV) injection, old versus young mouse microglia, cerebellar versus cortical microglia, perivascular macrophages (PVMs) relative to parenchymal microglia, or infiltrating macrophages versus brain-resident microglia. We tested each study’s set of DE genes for AD-enriched microglial expression in our SFG RNA-seq profiles. The mouse microglia aging profile, the PS2APP profile, and the PVM profile each showed statistically significant enrichment in AD versus control microglia (Figure 4B). However, as with the modular gene sets described earlier, such correlations were extremely subtle when viewed at the level of individual genes (Figure S5D).

Conversely, we looked at whether the DE genes identified in HAM (besides *APOE*, a well-known DAM gene) showed consistent trends in mouse models of neurodegeneration (Friedman et al., 2018; Orre et al., 2014; Wang et al., 2015), infection (Erny et al., 2015; Srinivasan et al., 2016), and aging (Grabert et al., 2016). Of the HAM-Up genes, only *PLXNC1*, *CD44*, *SMIM3*, and *ADAM8* were frequently though modestly increased in neurodegenerative mouse models (Figure S6). Of the HAM-Down genes, only *SERPINF1* showed consistent reduction in these models.

Altogether, the comparisons of AD microglia profiles with diverse mouse microglia profiles indicated that the HAM profile bore little resemblance to the DAM profile observed in mouse models of neurodegeneration or to other mouse microglia activation profiles. We next turned our attention to comparisons with published human microglia expression profiles.

### AD Microglia Display an Enhanced Human Aging Phenotype

**Figure 5.**
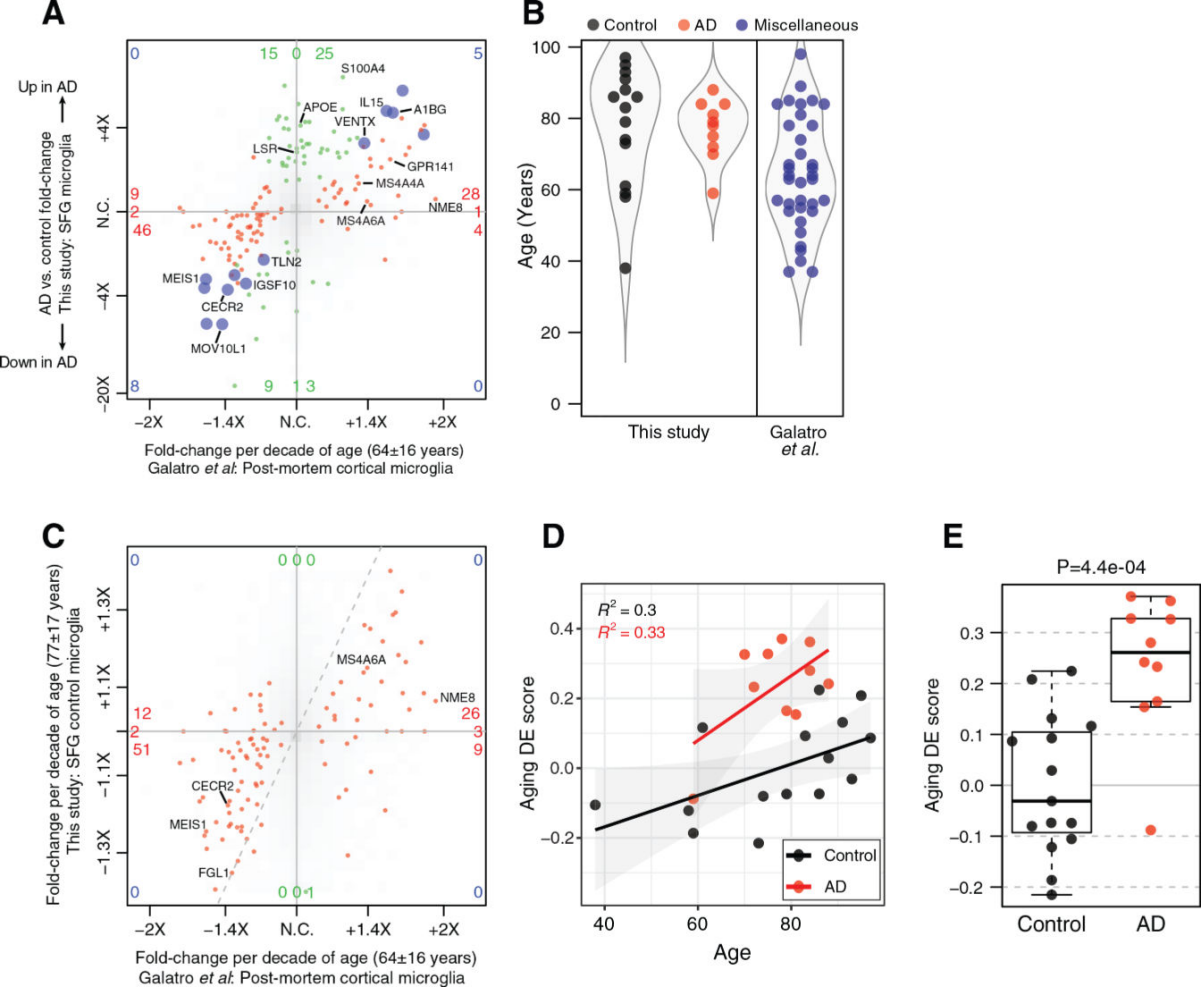
AD-Associated HAM Profile Overlaps Substantially with Age-Related DE Patterns in Human Microglia (A) 4-way DE plot (analogous to Figure 3B) shows age-related DE from Galatro et al. (2017) on the x axis and AD-related DE on the y axis. Color indicates p ≤ 0.05 significance with aging only (red), with AD only (green), or with both (blue). Most red genes, DE with age, trended in a consistent direction with AD versus control microglia (bottom-left and top-right quadrants), indicating that AD microglia exhibit enhanced aging. The green genes, including *APOE*, indicate an AD-related signature that is distinct from DE of normal aging. (B) Distribution of subject ages in both studies. (C) Previously reported DE pattern in normal, aged human microglia is recapitulated in control subjects of this study. The 4-way plot shows age-related DE from Galatro et al. (2017)’s dataset on the x axis, as in (A), and age-related DE from this study’s control SFG microglia profiles on the y axis. Genes in red met an adjusted p ≤ 0.05 cutoff in Galatro et al. (2017); other genes are shown as a smoothed density in shades of gray. No DE genes from Galatro et al. (2017) met the p ≤ 0.05 cutoff for age-related DE in our dataset, but most trended in a consistent direction (bottom-left and top-right quadrants). The lack of statistical significance and muted fold changes in our study may resultfromfar fewersamples and our samples coming mainly from subjects. (D) Aging DE score was calculated for each SFG microglia sample in our study—a signed average of the age-related DE genes from Galatro et al. (2017). Regression lines show the increasing trend of this score in both diagnosis groups with age, as well as the elevated score in the AD group relative to controls of similar ages. (E) Aging DE score is elevated in AD microglia relative to controls. y coordinates as in (D); p value, t test.

**Figure 6.**
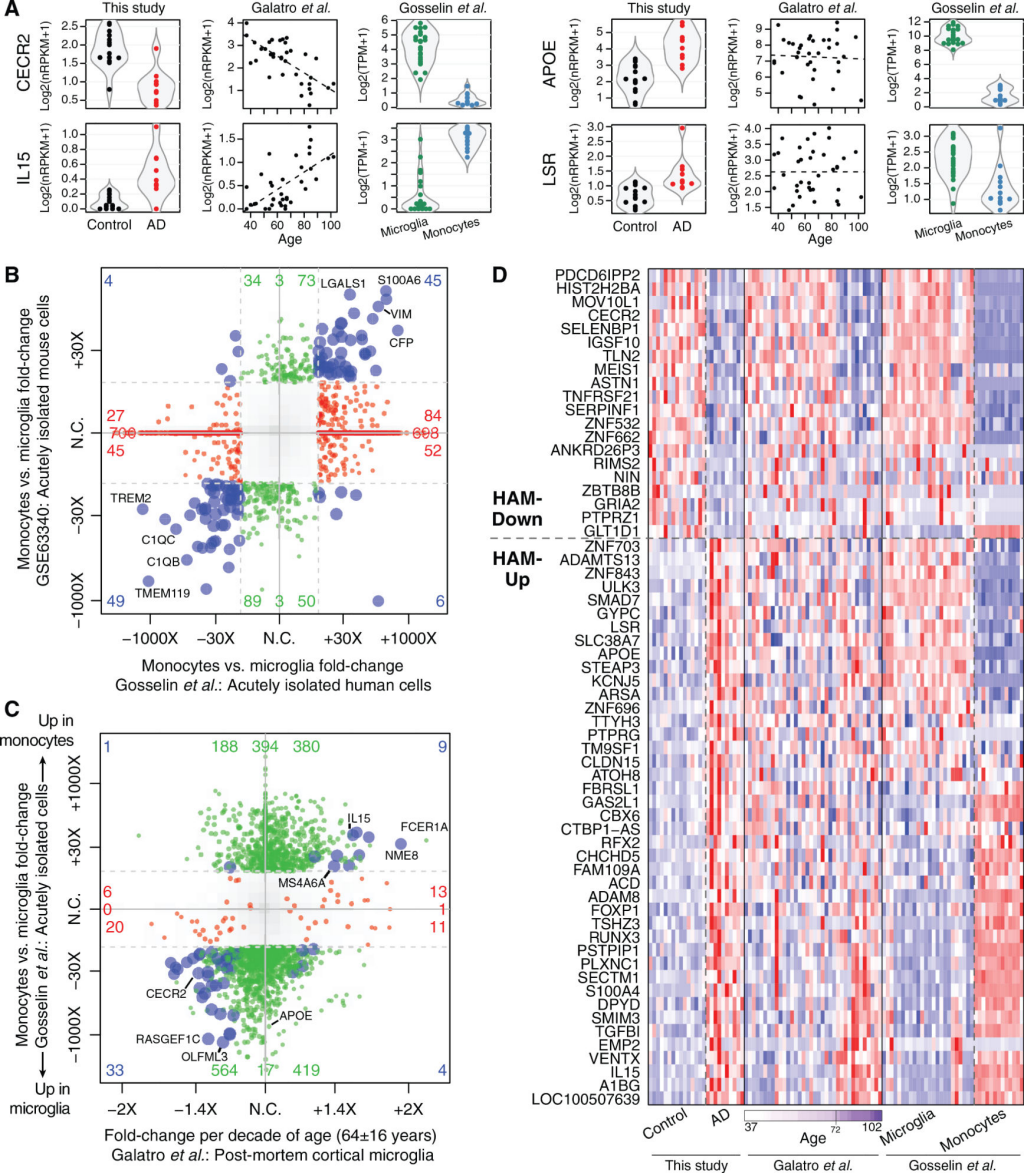
Monocyte-Enriched Genes May Contribute to Both Late Aging and AD Microglial Signatures (A) Example gene expression plots. Each point shows the expression of the indicated gene in a single sample in one of the three studies. In the middle column (Galatro et al., 2017), the dashed line indicates the best linear fit. (B) Monocyte DE profiles relative to microglia are similar in human and mouse studies. The 4-way plot is similar to Figure 3B but with DE genes between monocyte and microglia profiles shown with human and mouse studies on the x and y axes, respectively. (C) Many DE changes elevated or depleted in aged human microglia (x axis) are also elevated or depleted, respectively, in blood monocytes relative to microglia (y axis). The 4-way plot shows DE genes with p ≤ 0.05 in the aging study colored red, DE genes with p ≤ 0.05 and fold change ≥ 8 between monocytes and microglia colored green, and DE genes that meet both criteria colored blue. (D) Heatmap of DE genes from the HAM profile in three datasets. Gene ordering was based on the direction of change in this study and then by effect size (fold change per decade) in aging. The subset of HAM-Down genes that show reduced expression in aged microglia generally shows higher expression in microglia than in monocytes. The subset of HAM-Up genes that show increased expression in aged microglia generally shows higher expression in monocytes than in microglia.

Galatro et al. (2017) determined age-related changes in human microglial gene expression by sequencing RNA of microglia freshly sorted from post-mortem subjects spanning an age range of over six decades. In contrast to the preceding comparisons with mouse microglial datasets, the relationship between the HAM profile and the age-related DE in human microglia was striking. Most genes with higher expression in microglia from older subjects, including *IL15* and the candidate AD risk genes *MS4A6A*, *MS4A4A*, *NME8*, and *GPR141*, trended toward elevated expression in AD relative to control microglia; conversely, genes with lower expression in microglia from older subjects, like *CECR2*, tended to be reduced in AD microglia (Figures 5A and 6A). This did not result from differences in age between our AD and control subjects (Figure 5B).

We examined whether the age-related DE genes from Galatro et al. (2017) also showed a relationship with age in our dataset. Despite our dataset only including 15 control profiles, mostly from older subjects, we observed a clear tendency for genes whose expression changed with age in Galatro et al. (2017)’s dataset to show the same direction of age-related change among our control microglia samples (Figure 5C), validating our SFG RNA-seq profiles.

We used the age-related changes from Galatro et al. (2017)’s dataset to assign age-related DE scores to each of our SFG microglia profiles. When these scores were plotted against the subjects’ ages, we observed a positive correlation within both control and AD subject groups (Figure 5D), and the age-related DE scores for AD microglia as a group were significantly higher than the scores for the control group (Figure 5E). However, most AD-related DE genes from our dataset, including *APOE* and *LSR*, showed no relationship with age in microglia (Figures 5A and 6A). These data indicated that the HAM profile in AD microglia reflected a mixture of an enhanced aging process and an age-independent, disease-related activation process.

We analyzed another human dataset, from Gosselin et al. (2017), of microglia expression profiles obtained from fresh surgical tissue (all from subjects < 20 years old) and blood monocytes from the same subjects. DE genes between monocytes and microglia correlated reasonably well between human and mouse (Lavin et al., 2014) datasets (Figure 6B). Comparing the two human datasets, we saw some correlation between age-associated and microglia/monocyte DE: many genes with higher expression in younger subjects in Galatro et al. (2017)’s dataset, like *CECR2*, were microglia enriched in Gosselin et al. (2017)’s dataset; conversely, many genes with elevated expression in older subjects, like *IL15*, were monocyte enriched (Figures 6A and 6C). Viewing a heatmap of DE genes from the HAM profile across the three datasets, we saw that most HAM genes exhibiting age-related DE in the Galatro et al. (2017) study showed corresponding expression changes in monocytes relative to microglia (Figure 6D). These comparisons may suggest that some age-related changes in microglial gene expression could result from an increased presence of brain-infiltrating peripheral monocytes/macrophages in aged subjects and that infiltration by these cells is enhanced in AD. Alternatively, these changes could simply reflect microglial transcriptional modulation toward a state that bears some resemblance to monocyte profiles as subjects age, with that aspect more pronounced in AD subjects.

### HAM, DAM, and Aging Comparisons in Human AD, Xenograft AD, and Human MS Tissues

**Figure 7.**
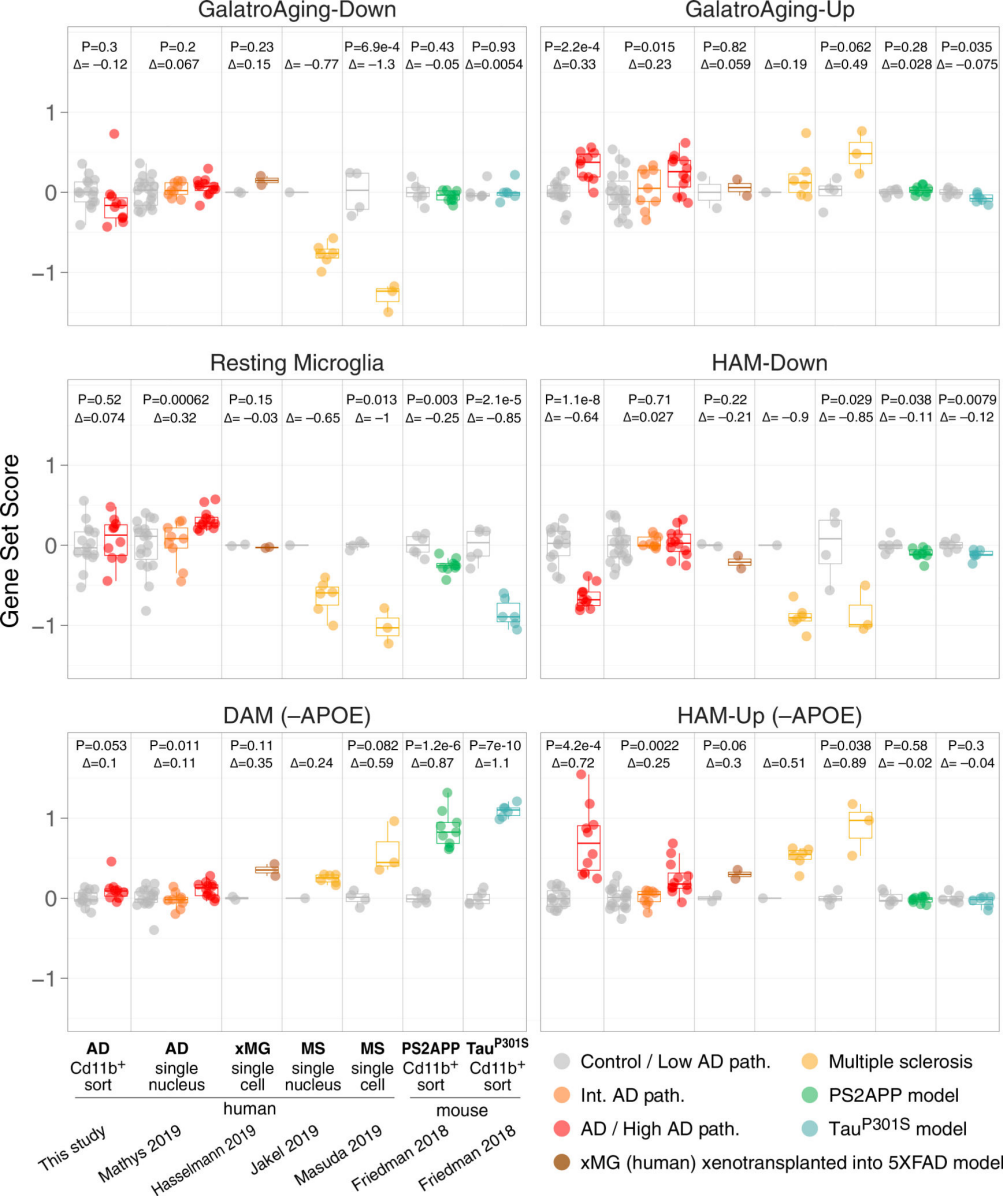
HAM Signature Is Elevated in Multiple Neurodegenerative Settings, whereas DAM Response Is Weaker in AD Microglia Control-centered scores (log2 scale) for the indicated gene sets were calculated for each sample in the indicated datasets. For snRNA-seq datasets (Mathys et al., 2019, frozen AD tissues, syn18485175; Jä kel et al., 2019, frozen MS tissues, GEO: GSE118257) and scRNA-seq datasets (Masuda et al., 2019, freshly resected MS lesions, GEO: GSE124335; Hasselmann et al., 2019, human induced pluripotent stem cell [iPSC]-derived xMG into 5xFAD mouse brains, GEO: GSE133433), each datapoint represents a pseudobulk microglia profile from pooling individual nuclei/cells from a given subject. (See Data S4, panels 2 and 3, for definitions of microglia clusters used to generate pseudobulk profiles from sn/scRNA-seq datasets.) Other datasets are bulk-sorted brain myeloid cells from frozen AD tissues (this study, GEO: GSE125050) or fresh mouse model tissues (PS2APP b-amyloid and PS19 Tau-P301S models, GEO: GSE89482 and GSE93180). D, log2 fold change of group means; p values from t test. For syn18485175, t test and D were between low- and high-pathology groups. The p value was omitted for GEO: GSE118257, because only one control sample was available (see STAR Methods). See STAR Methods (Gene Set Analysis section) for gene lists and Figure S7 for depictions of individual DE genes across studies.

Finally, we analyzed four recent datasets for evidence of HAM, DAM, or age-related activation states in human microglia scRNA-seq or snRNA-seq profiles obtained from AD tissues (syn18485175 at synapse.org) (Mathys et al., 2019), multiple sclerosis (MS) lesions (GEO: GSE124335 and GSE118257) (Jäkel et al., 2019; Masuda et al., 2019), and cells xenotransplanted into the mouse 5xFAD model (GEO: GSE133433) (Hasselmann et al., 2019). (See Data S4, panels 2 and 3, for t-distributed stochastic neighbor embedding [tSNE] plots, definition of myeloid cell clusters, and coloring by gene set scores.) For each subject, we aggregated cells from the microglial cluster into a single pseudo-bulk expression profile (see STAR Methods) and then scored the pseudobulk profiles for expression of the mouse DAM (neurodegeneration-related module; Friedman et al., 2018) gene set, our HAM-Up and HAM-Down gene sets, the human Aging-Up and Aging-Down gene sets (Galatro et al., 2017), and the mouse resting or homeostatic microglia module (Friedman et al., 2018) (Figure 7). We excluded *APOE* from the HAM-Up and DAM gene sets for these analyses so that the signal strengths for each gene set could be compared using only distinct features.

We detected increased expression of the HAM-Up gene set in microglial nuclei from tissues with high AD pathology (Figure 7). This increase was more substantial than the increased expression of the DAM gene set, which was marginal, thus corroborating our analyses in whole-tissue RNA profiles (Figure 3D) and SFG-sorted microglia profiles (Figure 4A). As a point of reference, sorted mouse microglia from the PS2APP and Tau-P301S models showed strong DAM induction and no changes in the HAM-Up gene set. The microglial nuclei from high AD pathology tissues also showed increased expression of the Aging-Up gene set. We did not detect reduced expression of the HAM-Down or Aging-Down gene sets in AD microglial nuclei, perhaps because most of these genes already have low expression and thus are poorly represented in the snRNA-seq data due to extensive gene dropout (Figure S7A), making their further downregulation in AD difficult to detect using this approach. Overall, our analysis of the snRNA-seq dataset confirmed that the DE we observed in our sorted CD11b^+^ cell population from AD tissues occurred within the microglial compartment, not in minor populations of co-purifying CD11b^+^ cells.

Interestingly, induction of DAM genes was stronger in human xenotransplanted microglia (xMG) in 5xFAD mouse brains, and in microglia from MS lesions, than it was in AD microglia (Figure 7). Thus, human microglia are capable of responding in a DAM-like manner, but for some reason this response is blunted in AD patients (at least in the disease stages we examined). For instance, *GPNMB* upregulation was robust in xMG and MS cells, but in AD microglia, it was meager or absent (Figure S7B). All components of the HAM profile—elevated HAM-Up and Aging-Up scores and reduced HAM-Down and Aging-Down scores— were clearly represented in microglia from MS lesions, usually with larger effect sizes and lower p values than the DAM scores in the same cells (Figure 7). In contrast, human xMG from 5xFAD mouse brains displayed similar extents of induction for HAM-Up and DAM gene sets, although no changes were observed in the Aging-Up and Aging-Down gene sets (Figure 7). This suggests that the enhanced aging profiles we observed in AD microglia are not a direct response to amyloid pathology.

Surprisingly, expression of the resting microglia module defined in mouse microglia was not reduced in microglia from AD tissues (it increased in the snRNA-seq dataset) or even in human xMG from 5xFAD mouse brains. In contrast,it was strongly reduced in microglia from MS lesions, being reduced to a similar or even greater extent than in mouse microglia from the Tau-P301S or PS2APP models, respectively (Figure 7). Considering the perforations in blood-brain-barrier integrity known to occur in MS, the apparent reduction in homeostatic gene expression observed in microglia from MS lesions may reflect infiltration of peripheral myeloid cells in which expression of this module is already low.

To further understand these consistent trends in gene set scores, we examined gene-by-gene concordance between the AD datasets. Differences in fold-change profiles in xMG and HAM were perhaps not surprising given the differences in context (top and bottom panels of Figure S7A). However, many genes detected as DE in our study did not replicate in the snRNA-seq profiles (middle panel), perhaps because of low detection in that study (gray) but also demonstrating that more studies are needed to elucidate the microglial response in HAM. Despite this, we identified several genes consistently upregulated across multiple datasets (see examples in Figure S7B).

## DISCUSSION

Here we have addressed the question of whether expression profiles from mouse AD models reflect activation states observed in HAM by employing a method for prospective isolation of defined cell types from frozen brain tissues that allowed us to survey ~100,000 microglial cells per tissue sample by RNA-seq. Unlike recent efforts to profile bulk-sorted microglia from freshly obtained AD tissue samples (Olah et al., 2018) or to profile microglia and other cell types from frozen tissue samples using snRNA-seq (Mathys et al., 2019), our approach allowed us to sample a suitably large number of tissues with known histopathological characteristics while obtaining broad coverage of the transcriptome. Though not affording single-cell resolution, this enabled more identification of DE genes and facilitated more substantive cross-comparisons with other datasets than the other methods.

The DE profile we observed in HAM (the HAM profile) was almost entirely distinct from the DAM profile defined in mouse models. Initially, we could not exclude that our experimental methods for tissue dissociation, labeling, and sorting precluded the detection of human microglia with DAM-like activation, but further analyses alleviated this concern. First, the HAM signal was clearly stronger than the DAM signal in AD whole-tissue RNA profiles. Second, the HAM signal was stronger than the DAM signal in snRNA-seq profiles from both AD tissues and MS lesions. Third, we did not observe instances of DAM^+^ nuclei clustering separately from HAM^+^ nuclei in the snRNA-seq datasets; instead, these datasets revealed that to whatever extent the DAM signal was induced, it occurred in the same nuclei in which the HAM signal was detected (see Data S4, panel 2). That the DAM activation state—generally considered protective in mouse neurodegeneration models—was more readily observed in microglia from MS lesions and in xMG from 5xFAD mouse brains suggests that its relative lack of induction in AD microglia may be a unique aspect of late-onset AD.

Despite the dissimilarity between DAM and HAM signatures, one qualitative similarity emerges. Just as DAM genes induced in neurodegenerative mouse models overlap with those induced by natural aging (Friedman et al., 2018; Holtman et al., 2015), so do many HAM genes induced in human AD tissues (Figure 5A), though the genes involved are distinct between species (Galatro etal.,2017).Another emergingthemeinmousemodel literature is the involvement of some DAM genes (such as *Apoe*, *Ch25h*, *Lpl*, *Ctsb*,and *Atp6v0d2*)in lipid andlysosomalbiology and theinduc-tion of DAM gene expression by lipid pathologies such as demyelination (Nugent et al., 2020; Poliani et al., 2015) and atherosclerosis (Cochain et al., 2018; Kim et al., 2018). In our data, in addition to *APOE*, we found that the lipoprotein receptor *LSR* and the lysosomal enzyme *ARSA*—a gene in which homozygous mutations cause metachromatic leukodystrophy (Cesani et al., 2016)—were elevated in HAM. Therefore, another possible simi-laritybetweenDAMandHAMprofilescouldbetheinvolvementof lipid/lysosomal biology-associated genes.Several genesassoci-ated with AD incidence (*APOE*, *CLU*, *ABCA7*, *SORL1*, *INPP5D*, and *PLCG2*)(Jansen et al., 2019; Kunkle et al., 2019; Marioni et al., 2018) also function in lipid transport or signaling.

Why are the HAM and DAM gene signatures so different? One explanation could be intrinsic differences in human versus mouse innate immune responses, but the activation of many DAM genes in MS lesions and in xMG from 5xFAD mouse brains suggests this is not the only reason. Another explanation could be the different stages of disease being analyzed, with mouse β-amyloid models perhaps representing early-stage AD with amyloid deposits present but preceding neurodegeneration. However, if this were the main reason, we might expect to see mouse DAM genes elevated in tissues in early Braak stages and decreased in tissues in later Braak stages, but we have not observed such trends in whole-tissue RNA profiles. A third explanation for the dissimilarity could be that the DAM activation state in b-amyloid models is a protective response by healthy microglia (Keren-Shaul et al., 2017), whereas genetic and histological findings suggest that human AD involves impairments in microglial activation (Hansen et al., 2018; Streit et al., 2009). Additional profiles with increased cellular resolution for various AD stages and brain regions, different neurodegenerative diseases, and additional disease models that incorporate human microglial cells will shed further light on how the HAM profile relates to mechanisms of AD protection or pathogenesis.

## STAR★METHODS

### RESOURCE AVAILABILITY

#### Lead Contact

Further information and requests for resources should be directed to and will be fulfilled by the Lead Contact, Brad Friedman (friedman.brad@gene.com).

#### Materials Availability

This study did not generate new unique reagents.

#### Data and Code Availability

All new RNA-Seq data described in this study are available from the GEO/SRA repository: GSE125050 (sorted cell RNA-Seq from AD and control SFG) and GSE125583 (bulk tissue RNA expression from FuG of AD and Control subjects). This study did not generate any new software; questions about data analysis should be directed to the Lead Contact, Brad Friedman (friedman.brad@gene.com).

### EXPERIMENTAL MODEL AND SUBJECT DETAILS

Frozen superior frontal gyrus and fusiform gyrus tissue blocks and pathology/clinical reports, including age, sex, diagnosis, and Braak stage, were obtained from the Banner Sun Health Research Institute Brain and Body Donation Program in accordance with institutional review boards and policies at both Genentech and Banner Sun Health Research Institute. All samples obtained from Banner Sun Health Research Institute were stored at 80 C until the time of processing.

All subjects had been characterized clinically and neuropathologically by the Arizona Study of Aging and Neurodegenerative Disease/Brain and Body Donation Program (Beach et al., 2015). All AD subjects were clinically diagnosed with AD in life and brains were neuropathologically confirmed to have "frequent" CERAD neuritic plaque densities (Mirra et al., 1991) and Braak score V or VI ( Braak and Braak, 1991). Controls did not have dementia, AD or other neurological disease diagnoses in life.

For sorted cell cohort (GSE125050), controls had either "zero" or "sparse" CERAD neuritic plaque densities, and mostly had Braak scores ranging from 0 to III (median II). One control subject was designated Braak stage IV due to slight tau pathology in the amygdala, and one control subject was diagnosed post-mortem with "argyrophilic grain disease."

**Table:** 

	All Subjects			Subjects with QC-passing Myeloid Profiles		
		
	Control	AD	p*	Control	AD	p*

N	21	21		15	10	

Male	13 (62%)	10 (48%)	0.536	10 (67%)	5 (50%)	0.442

ApoE4+	0 (0%)	9 (43%)	0.00132	0 (0%)	6 (60%)	0.00119

Age	80 (71 –88)	79 (72–84)	0.943	83 (67–89.5)	78.5 (72.8–83.2)	0.938

PMI	3 (2.5–3.25)	3 (2.33–3.08)	0.519	3 (2.75–3.2)	2.92 (2.2–3)	0.619

Last MMSE	28.5 (27.8–29)	1 (0–4)	6.52E-27	28.5 (27.8–29.2)	0.5 (0–5.5)	5.39E-11

* p values for Sex (Male) and ApoE4 status from Fisher’s Exact Test, others from Student’s t test. Median and interquartile range shown for Age/Post-mortem interval (PMI)/Last Mini-Mental State Exam (MMSE).

Linear model testing as well as visual exploration revealed no significant correlation between PMI and any of the other variables (diagnosis, see also Figure S2C PMI panel; sex; ApoE4 status; age; or Last MMSE).

Whole tissue studies cohorts were as follows:

**Table:** 

	GSE95587 (previously published)			GSE125583 (new subjects in this study)		
		
	Control	AD	P*	Control	AD	P*

N	33	84		42	158	

Male	23 (70%)	42 (50%)	0.0644	19 (45%)	86 (55%)	0.3

ApoE4+	8 (24%)	38 (45%)	0.0574	5 (12%)	85 (54%)	5.29E-07

Age	82 (80–90)	87 (81–91)	0.471	89 (84.2–91)	84 (77–88)	7.45E-06

Last MMSE	29 (28–29)	17 (7–22)	2.10E-23	28.5 (27–30)	14 (6–21)	1.06E-47

* P values for Sex (Male) and ApoE4 status from Fisher’s Exact Test, others from Student’s t test. Median and interquartile range shown for Age and Last MMSE.

Although age was not well controlled in the new cohort, the direction of difference was anti-conservative, with the AD cases on average about a half decade younger.

### METHOD DETAILS

#### Tissue processing, library preparation, and RNA-Seq for whole tissue RNA studies

For whole tissue RNA studies (GSE125583), frozen tissue was sectioned in approximately 8 slices 40 mm thick and stored at 80 C. Tissue was homogenized in 1 mL QIAzol with 5 mm stainless steel beads using a Tissuelyzer (20 Hz for 4 min). After homogenization, 200 μL of choloroform were added to the cleared lysate (1 min at 12,000 r*cf.* at 4°C), vigorously shook and incubated at room temperature 2–3 min. Samples were centrifuged for 15 min at 12,000 r*cf.* at 4°C and the upper aqueous phase was transferred to a new tube. RNA was extracted using QIAGEN miRNeasy mini columns, yielding samples with RNA integrity (RIN) scores averaging 6.5. Standard polyA-selected Illumina RNA-Seq analysis was performed as described (Srinivasan et al., 2016) on samples with RNA integrity (RIN) scores at least 5 and post-mortem intervals (PMIs) no greater than 5 hr. Of 289 total samples, 89 were from subjects that had already been profiled in our previous study, GSE95587. These are available in GSE125583 and marked therein as duplicated in GSE95587. These samples, which came from new fusiform gyrus tissue blocks, showed very similar sample-by-sample DE profiles as the corresponding samples from the same subjects in GSE95587 (Figure S4A), but were omitted in all other analyses associated with this manuscript to avoid overlap between the two datasets (see Figures 3D, S4B, and S4C; Data S2 and S3; and website).

#### Tissue processing, library preparation, and RNA-Seq for sorted cell studies

For sorted cell studies, frozen samples were opened on dry ice and a 100–200 mg portion was excised. The excised portion was thawed in ice-cold Hibernate A and minced on a cold block with a pre-chilled razor. Minced SFG samples included both gray and white matter, while only gray matter from FuG was used for mincing since gray matter atrophy was pronounced in FuG from AD subjects and we did not want differences between AD and control microglia to be dominated by potential differences between white matter and gray matter microglia. (For sixteen SFG samples, excess minced tissue fragments were refrozen and stored for a later attempt to repeat the entire sorting and RNA-Seq procedure from the same brain region—see QC section below.)

Minced tissue was transferred to a 2 mL round-bottom tube with cold 1.6 mL of Accutase and incubated 20–30 minutes on a rotator at 4°C, mechanically dissociated/triturated by pipetting, centrifuged, resuspended, and ethanol-fixed for 10 minutes on ice as previously described (Srinivasan et al., 2016). Cells were washed briefly and incubated with anti-CD11b APC (Millipore MABF366), anti-GFAP PE (BD PharMingen 561483), anti-NeuN AlexaFluor488 (Millipore MAB377X), anti-CD31 PE-Cy7 (BD PharMingen 563651), and Human Fc Block (BD PharMingen 564220) for 20 minutes at 4°C with sample rotation. Cells were centrifuged at 2,000 r*cf.* for 2 minutes and briefly washed prior to DAPI (1 mg/ml stock) being added at 1:1,000 followed by FACS sorting on ARIA sorters. Only DAPI+ singlet cell bodies were collected, and each cell population of interest was gated to be negative for all the other antibody marker channels. Samples were generally processed in pairs, with one AD and one control sample. While each human sample was unique and gating was occasionally fine-tuned, samples generally separated based on the same broad FACS gates. (We did not attempt to distinguish CD45low parenchymal microglia from CD45high peripheral/perivascular macrophages primarily for biological reasons since we did not want to exclude activated microglia which often display elevated CD45 reactivity, but also for technical reasons since we have not found a CD45 stain compatible with ethanol fixation.)

Typical cell numbers collected were 100K CD11b+ cells, 40K GFAP+ cells, 10K CD31+ cells, and 400K NeuN+ cells. FACS-isolated cell populations were spun at 5,000 r*cf.* for 5 minutes and resuspended in 0.35 mL Buffer RLT from QIAGEN RNeasy Micro kit. Lysed samples were stored at 80 C until all samples for a given brain region were sorted. Each cell type was then processed for RNA purification as a single batch. Typical RNA yields were 1 μg for neurons, 25 ng for microglia and astrocytes, and 5 ng for endothelial cells. RNA integrity (RIN) and concentration were determined by 2100 Bioanalyzer (Agilent Technologies). RIN scores for all cell types were typically between 1 and 3. Total RNA extracted from sorted cell populations was subjected to Fluidigm qPCR assay which yielded reliable cell-specific gene expression data, despite poor RNA quality resulting from post-mortem status, freeze/thaw process and fixation. In addition to the methods for dissociation and immunolabeling described above, we also attempted dissociation techniques involving trypsin or papain at 37 C, psychrophilic proteases at 4°C, longer Accutase treatment periods, automated mechanical dissociation instead of pipetting, other fixatives besides ethanol, labeling and sorting of non-fixed cells for cell types with surface markers (CD11b and CD31), and antibodies for alternative cell type markers. None of these attempts were as good as the method described above in terms of cell yield and RNA recovery.

Given the highly fragmented condition of our sorted cell RNA preps, we chose the NuGEN Ovation RNA-Seq System V2 kit for cDNA synthesis since it uses random oligos for cDNA priming. We knew this would result in high percentages of intronic and non-coding RNA reads, but our priority was to sample across all exons instead of having an extreme 30 bias and reduced complexity in our library. (Only exonic reads were counted toward nRPKM values.) Generated cDNA was sheared to 150–200bp size using LE220 ultrasonicator (Covaris). Following shearing, the size of cDNA was determined by Bioanalyzer DNA 1000 Kit (Agilent) and quantity was determined by Qubit dsDNA BR Assay (Life Technologies). Sheared cDNA was subjected to library generation, starting at end repair step, using Illumina’s TruSeq RNA Sample Preparation Kit v2 (Illumina). Size of the libraries was confirmed using 4200 TapeStation and High Sensitivity D1K screen tape (Agilent Technologies) and their concentration was determined using KAPA Library Quantification kits. The libraries were multiplexed within cell types and then sequenced on Illumina HiSeq2500 (Illumina) to generate 50M of single end 50bp reads.

### QUANTIFICATION AND STATISTICAL ANALYSIS

#### RNA-Seq data processing and QC for whole tissue samples and bulk cell type samples

Sorted cell and whole tissue RNA-Seq data were analyzed using the GSNAP aligner and HTSeqGenie as described (Friedman et al., 2018), except as follows. For Gosselin et al. (2017) (phs001373.v1.p1, human monocytes and microglia) we did not have access to the raw FASTQ files, so we used the author-provided tables of counts and TPM values. For ROSMAP-DLPFC we downloaded the file ROSMAP_RNAseq_FPKM_gene_plots_1_to_6_normalized.tsv from the synapse.org website, in order to take advantage of the batch normalization that the authors already applied. We did not use the samples from batches 7 and 8 since, despite restricting to the batch-normalized values, we still saw very strong clustering of these two batches separately from the first 6 on PCA. “Pass” or “Fail” status for our sorted cell RNA-Seq profiles was determined primarily using tSNE analysis (perplexity = 14, theta = 0.4) colored by cell type to visualize how profiles clustered (Figure S2A). tSNE clustering of profiles was generally confirmed by sample similarity heatmaps (not shown). Interpretation of tSNE clusters was informed by gene versus sample heatmaps (similar to the heatmap in Figure S2B but with unbiased hierarchical clustering of the 500 most variable genes, and blinded to AD diagnosis), which enabled us to see which tSNE clusters contained libraries with neat cell type-specific expression profiles and which clusters contained libraries with degenerate features including reduced specificity of cell type expression markers (see Figure S2B). Compared to "Pass" libraries, "Fail" libraries generally showed higher percentages of intergenic reads and lower percentages of exonic and intronic reads (see Figure S2C). We discarded 1/43 neuron libraries, 19/38 astrocyte libraries, 14/41 endothelial cell libraries, and 18/43 microglia libraries from original frozen tissues, and 16/16 microglia libraries from twice frozen tissues (which underscored the liabilities of the freeze-thaw process).

Principal Component Analysis (Figure 1C) was performed on Z-score normalized matrix of 1000 most variable genes by IQR using the R function prcomp().

#### Differential expression (DE) analysis for bulk-sorted cells

DE between AD and controls for this study’s sorted cell populations was first attempted using voom+limma, which identified only 12 DE genes (adjusted p ≤ 0.05) in myeloid cells and none in the other cell types. We then used DESeq2 instead (adjusted p ≤ 0.05), but we used the DESeq2-provided Cook’s distances to filter out genes likely driven by outlier samples. Any gene for which the Cook’s distance was greater than the α = 0.01 critical value of the *F* distribution was omitted from our DE genes lists. The Cook’s distance filter eliminated 6/10 neuronal DE genes, 9/75 myeloid DE genes, and 382/517 endothelial DE genes from consideration, leaving 4 DE genes in neurons, 66 in myeloid cells, and 135 in endothelial cells. The absence of any voom+limma hits for neurons and endothelial cells, the high fraction of DESeq2 hits driven by outliers in these two cell types, and the lack of other human AD datasets available at the time for cross-comparison led us to set these cell types aside (taking a conservative position) and focus on the whether the changes in myeloid cells could be validated. In the myeloid cells, 11/12 DE genes identified by voom+limma were also identified by DESeq2, with *CD44* being the only exception (p = 0.113 in DESeq2). We included *CD44* in our panel of genes tested by qPCR in FuG myeloid cell sorts, and it was again increased in the AD samples (unadjusted p = 0.041), so we consider its DE to be genuine though we did not include it in our HAM-Up gene set analyses, other than visualizing it in Figure S6A.

Our analysis of Galatro et al. (2017) was performed using DESeq2 (adjusted P value ≤ 0.05, maximum Cook’s P value ≥ 0.01). For Galatro et al. (2017) the ages of the subjects were taken from their supplemental table rather than GEO (these differed only for the sample GSM2631906), and the DE analysis was simply the linear model ~Age, only using the samples with tissue = "Microglia." For Gosselin et al. (2017), DE between microglia and monocytes was performed using DESeq2 using only the samples with Cultur-eStatus = "ExVivo."

#### Single-cell/nucleus RNA-Seq analysis

For Mathys et al. (2019) (syn18485175, human AD snRNA-Seq), count tables provided on synapse.org website were used as input. Gene symbols, or, if necessary, aliases, were used to map onto our internal gene annotation, based on Ensembl. NIA Reagan scores (for low, intermediate and high pathology) were obtained from Rush University via synapse.org. Analysis in this manuscript was limited to cells with the authors’ provided broad.cell.type = "Mic," although only the subset of these cells that we believe represent parenchymal microglia were used for the pseudobulk (see Data S4, panel 2). Total transcript number normalization was performed, dividing each gene expression value for a cell by a factor proportional to the total number of transcripts in that cell.

For Masuda etal. (2019) (GEO: GSE124335, scRNA-Seq ofCD45+ cells from fresh surgical resections of MS and controlpatients), we downloaded each of the 32 gene quantification files from the GSE124335 GEO record (file names like GSM3529822_MS_case1_ 3.coutt.csv.gz). These files each contained 192 columns corresponding to the cells of one batch, and one row per gene. The gene symbols were mapped onto IDs as described above. After this step cells with less than 800 total transcripts or greater than 30% mitochondrial transcripts were discarded, resulting in 1,738 QC-passing cells for analysis. Total transcript number normalization was performed as describe above.

For Jäkel et al. (2019) (GEO: GSE118257, snRNA-Seq of post-mortem MS and control brains) and Hasselmann et al. (2019) (GEO: GSE133433, scRNA-Seq of xMG in 5xFAD and non-diseased mouse brains), the single-nucleus/cell count tables were similarly downloaded from GEO and processed as above. For Jä kel et al. (2019) only nuclei with at least 400 total UMIs were taken for analysis, and for Hasselmann et al. (2019) only cells with log10(total UMIs) R 3.25 and at most 5% mitochondrial transcripts were taken.

R/Seurat was used to calculate PCA, tSNE coordinates and Louvain clustering for all of these studies. Cell IDs, tSNE coordinates, Seurat clusters, and interpretations of Seurat clusters for each cell visualized in Data S4, panels 2 and 3, and individual cell-level results in Data S5 (CSV file).

#### Pseudo-bulk analysis of sc/snRNA-Seq datasets

Pseudo-bulk datasets were derived from single-cell/single-nucleus datasets first by aggregating the cells of each sample of the same cell type. So, for *n* samples and *m* cell types there were *nm* total possible *pseudo-bulks* (that is, aggregates of cells of a single type from a single sample). If fewer than 10 cells of a particular type were present in a given sample then they were discarded, so the actual total number of pseudo-bulks was typically less than *nm*. A single "raw count" expression profile was created for each pseudobulk simply by adding the total number of UMIs for each gene across all the cells. This gave a gene-by-pseudobulk count matrix which was then normalized to a normalizedCount statistic using the estimateSizeFactors function from DESeq2, used for calculating gene set scores and visualizing gene expression, and for normalization factors for differential expression analysis. DE was performed on pseudobulk data-sets using voom+limma methods for bulk RNA-Seq.

To put this into more formal notation, let *n_ij_* be the raw UMI number of gene *i* in cell *j*. Let *s_j_* and *c_j_* indicate the sample and cell type, respectively, of cell *j*.

Then the pseudobulks are the set of pairs (*s*;*c*) of samples *s* and cell types *c* for which there are at least 10 cells *j* with (*s_j_*;*c_j_)* = (*s*;*c)*. The pseudobulk count matrix *B*, with rows indexed by genes and columns indexed by pseudobulks (that is, (*s*; *c)* pairs) is defined as

$$B_{i,sc}=\sum_{j:(S_{j},C_{j})=(S,C)}n_{ij}$$

The matrix *B* is then analyzed using the standard methods of bulk RNA-Seq.

#### Other covariates: Post-mortem interval, sex, APOE genotype

Differential expression analysis (Data S4, panel 1) revealed that the expression of about 80 genes was significantly increased in microglia from subjects with larger post-mortem interval (PMI). This seemed to be largely driven by elevated mitochondrial gene expression in a subset of the samples with large PMI. However, the distribution of PMI in our AD and control samples was similar (Figure S2C; Data S4, panel 1A, inset), there was no overlap between the AD-related DE genes and the PMI-associated genes, and adding PMI to our statistical model for AD-associated DE gave very similar results. Therefore, we did not include PMI in subsequent analyses. Sex-associated DE in microglia was almost entirely restricted to X and Y chromosome genes. For *APOE* genotype, we only detected one DE gene, *ACY3*, in AD microglia between *APOE-*e4 carriers versus non-carriers. It showed variable expression levels in the Controls (all non-carriers), so it may be a false positive.

#### Fluidigm qPCR analysis

qPCR data were collected as described (Srinivasan et al., 2016). Then, for each assay target, the maximum *C_t_* of quality > 0 was calculated. The *C_t_* value maxCt+0.5 was assigned to each assay that had *C_t_* larger than this value (including 999). All assays were performed in duplicate and the average of these two *C_t_* values was kept, except for twelve sample/assay pairs for which the difference was more than 2.82 (corresponding to a standard deviation of 2), which were discarded. Δ*C_t_* normalization was performed using global median (the median Ct value for all assays for a given sample) and differential expression between AD and control was performed using limma.

#### Gene set analysis

Gene Sets can be found in Data S3, as follows:

- Figures 1B and S2B: Cell type marker genes in column O "Barres Human cell Types."
- Figure 3D: "HAM-Up"/"HAM-Down" are the DE genes from this study, noted in column N. "DAM" are disease/damage-associated microglia genes, called "Neurodegeneration-Related" in column Q "Myeloid Activation (Coarse)." "LPS-Specific" genes are significantly induced in myeloid cells by LPS but not significantly changed in myeloid cells in response to LCMV, b-amyloid, Tau pathology, or SOD1G93A, in column R "Immune-Specific."
- Figure 4A and Table S1: Mouse-derived gene sets (left panel) in column Q "Myeloid Activation (Coarse)" except for "LPS-Specific" in column R "Immune-Specific." BrainMyeloid gene set contains the orthologs of the union of gene modules 2, 3, 5, 7 and 9 from previous publication (Friedman et al., 2018) (column T of that manuscript’s Data S4). These are genes elevated in microglia relative to infiltrating and peripheral macrophages but not so much relative to perivascular macrophages.
- Figure 4B: DE gene sets taken from our previous manuscript (Friedman et al., 2018)
- Figure 7: "GalatroAging-Down" and "GalatroAging-Up" are the genes DE with age (depicted in Figures 5A and 5C), with DE stats and adjusted p < 0.05 in columns CU-CW. "Resting Microglia" refers to the Microglia module genes annotated in column Q. Other gene sets described above, with *APOE* removed as indicated.
- Figure S4C: Same gene sets as Figure 3D, plus neuron and myeloid markers from Figure 1B
- Figures S5A–S5C: Same gene sets as Figure 4A
- Data S4, panels 2 and 3: Gene sets not described above are included in column P "ABA Mouse Cell types," column S "scRNA-Seq Characterization," and column T "FerritinCluster."

Gene set scores (Figures 3D, 4A, 7, S4B, and S4C; Data S4, panels 2 and 3) were calculated as described (Friedman et al., 2018). Briefly, gene expression values were first log-transformed and stabilized as Log2(nRPKM+1), or, for ROSMAP-DLPFC, Log2(normalized RSEM+1). Then the average log-scale expression values of the controls were subtracted out for each dataset to yield control-centered gene expression values. The gene set score for a sample was then calculated as the average over all genes in the set of the control-centered gene expression values. For DE scores (Figures 3C, 4B, 5D, 5E, S3A, and S4A) a similar method was used, but with a signed average: up genes were weighted by +1 and down genes by −1 to capture comparisons of both up and down genes in a single score.

In cases where gene set scores were presented in the same figure or analysis in a manner that suggested or required cross-project comparisons (Figures 3C, 3D, 7, S3A, and S4; Data S4, panels 2 and 3), gene sets were limited to those genes present in all studies compared.

Myeloid balancing (Figures 3D and S4B) of whole tissue RNA profiles was performed as described (Friedman et al., 2018). Briefly, for each dataset, samples were split into 20 bins of similar myeloid gene set scores. In each bin, control or AD samples were randomly discarded as needed to reduce differences in the ratio of AD to control samples across bins.

### ADDITIONAL RESOURCES

Brain Myeloid Landscape 2 Website: http://research-pub.gene.com/BrainMyeloidLandscape. This website updates our previously released resource at the same URL with the datasets described in this manuscript. Users can enter genes of interest and quickly see their differential expression across all of these brain myeloid-related datasets, as well as expression within the samples in each individual dataset.

## Supplementary Material

1

2

3

4

5

6

7

## Figures and Tables

**KEY RESOURCES TABLE:** 

REAGENT or RESOURCE	SOURCE	IDENTIFIER
Antibodies		
anti-CD11b APC	Millipore	MABF366; RRID:AB_2857951
anti-GFAR PE	BD PharMingen	561483; RRID:AB_10689630
anti-NeuN AlexaFluor488	Millipore	MAB377X; RRID:AB_2149209
anti-CD31 PE-Cy7	BD PharMingen	563651; RRID:AB_2738348
Chemicals, Peptides, and Recombinant Proteins		
Human Fc Block	BD PharMingen	564220
Deposited Data		
Sorted-cell RNA-Seq Data from AD and Control SFG	GEO	GSE125050
Bulk Tissue RNA-Seq Data from AD and Control FuG	GEO	GSE125583

## Highlights

- Transcriptomic data from FACS-sorted microglia from frozen AD and control samples
- APOE and other differentially expressed genes define the human Alzheimer’s microglia state
- Scant overlap with mouse models but strong enrichment of age-related changes
- Web resource to browse data: http://research-pub.gene.com/BrainMyeloidLandscape

## References

[R1] Beach TG, Adler CH, Sue LI, Serrano G, Shill HA, Walker DG, Lue L, Roher AE, Dugger BN, Maarouf C, (2015). Arizona Study of Aging and Neurodegenerative Disorders and Brain and Body Donation Program. Neuropathology 35, 354–389. 25619230 10.1111/neup.12189 PMC4593391

[R2] Bellenguez C, Grenier-Boley B, and Lambert JC (2020). Genetics of Alzheimer’s disease: where we are, and where we are going. Curr. Opin. Neurobiol 61, 40–48. 31863938 10.1016/j.conb.2019.11.024

[R3] Boulay AC, Saubamé a B, Adam N, Chasseigneaux S, Mazare N, Gilbert A, Bahin M, Bastianelli L, Blugeon C, Perrin S, (2017). Translation in astrocyte distal processes sets molecular heterogeneity at the glio-vascular interface. Cell Discov. 3, 17005. 28377822 10.1038/celldisc.2017.5 PMC5368712

[R4] Braak H, and Braak E (1991). Neuropathological stageing of Alzheimer-related changes. Acta Neuropathol. 82, 239–259. 1759558 10.1007/BF00308809

[R5] Cesani M, Lorioli L, Grossi S, Amico G, Fumagalli F, Spiga I, Filocamo M, and Biffi A (2016). Mutation Update of ARSA and PSAP Genes Causing Metachromatic Leukodystrophy. Hum. Mutat. 37, 16–27. 26462614 10.1002/humu.22919

[R6] Chang YT, Huang CW, Chen NC, Lin KJ, Huang SH, Chang WN, Hsu SW, Hsu CW, Chen HH, and Chang CC (2016). Hippocampal Amyloid Burden with Downstream Fusiform Gyrus Atrophy Correlate with Face Matching Task Scores in Early Stage Alzheimer’s Disease. Front. Aging Neuro-sci 8, 145. 10.3389/fnagi.2016.00145 PMC4911390 27378917

[R7] Cochain C, Vafadarnejad E, Arampatzi P, Pelisek J, Winkels H, Ley K, Wolf D, Saliba AE, and Zernecke A (2018). Single-Cell RNA-Seq Reveals the Transcriptional Landscape and Heterogeneity of Aortic Macrophages in Murine Atherosclerosis. Circ. Res. 122, 1661–1674. 29545365 10.1161/CIRCRESAHA.117.312509

[R8] De Jager PL, Ma Y, McCabe C, Xu J, Vardarajan BN, Felsky D, Klein HU, White CC, Peters MA, Lodgson B, (2018). A multi-omic atlas of the human frontal cortex for aging and Alzheimer’s disease research. Sci. Data 5, 180142. 30084846 10.1038/sdata.2018.142 PMC6080491

[R9] Deczkowska A, Keren-Shaul H, Weiner A, Colonna M, Schwartz M, and Amit I (2018). Disease-Associated Microglia: A Universal Immune Sensor of Neurodegeneration. Cell 173, 1073–1081. 29775591 10.1016/j.cell.2018.05.003

[R10] Del-Aguila JL, Li Z, Dube U, Mihindukulasuriya KA, Budde JP, Fernandez MV, Ibanez L, Bradley J, Wang F, Bergmann K, (2019). A single-nuclei RNA sequencing study of Mendelian and sporadic AD in the human brain. Alzheimers Res. Ther. 11,71. 31399126 10.1186/s13195-019-0524-x PMC6689177

[R11] du Boisgueheneuc F, Levy R, Volle E, Seassau M, Duffau H, Kinkingne-hun S, Samson Y, Zhang S, and Dubois B (2006). Functions of the left superior frontal gyrus in humans: a lesion study. Brain 129, 3315–3328. 16984899 10.1093/brain/awl244

[R12] Erny D, HrabedeAngelis AL, Jaitin D, Wieghofer P, Staszewski O, David E, Keren-Shaul H, Mahlakoiv T, Jakobshagen K, Buch T, (2015). Host microbiota constantly control maturation and function of microglia in the CNS. Nat. Neurosci. 18, 965–977. 26030851 10.1038/nn.4030 PMC5528863

[R13] Friedman BA, Srinivasan K, Ayalon G, Meilandt WJ, Lin H, Huntley MA, Cao Y, Lee SH, Haddick PCG, Ngu H, (2018). Diverse Brain Myeloid Expression Profiles Reveal Distinct Microglial Activation States and Aspects of Alzheimer’s Disease Not Evident in Mouse Models. Cell Rep 22, 832–847. 29346778 10.1016/j.celrep.2017.12.066

[R14] Galatro TF, Holtman IR, Lerario AM, Vainchtein ID, Brouwer N, Sola PR, Veras MM, Pereira TF, Leite REP, Mö ller T, (2017). Transcriptomic analysis of purified human cortical microglia reveals age-associated changes. Nat. Neurosci. 20, 1162–1171. 28671693 10.1038/nn.4597

[R15] Gosselin D, Skola D, Coufal NG, Holtman IR, Schlachetzki JCM, Sajti E, Jaeger BN, O’Connor C, Fitzpatrick C, Pasillas MP, (2017). An environment-dependent transcriptional network specifies human microglia identity. Science 356, eaal3222. 28546318 10.1126/science.aal3222 PMC5858585

[R16] Grabert K, Michoel T, Karavolos MH, Clohisey S, Baillie JK, Stevens MP, Freeman TC, Summers KM, and McColl BW (2016). Microglial brain region-dependent diversity and selective regional sensitivities to aging. Nat. Neurosci. 19, 504–516. 26780511 10.1038/nn.4222 PMC4768346

[R17] Hansen DV, Hanson JE, and Sheng M (2018). Microglia in Alzheimer’s disease. J. Cell Biol. 217, 459–472. 29196460 10.1083/jcb.201709069 PMC5800817

[R18] Hasselmann J, Coburn MA, England W, Figueroa Velez DX, Kiani Sha-bestari S, Tu CH, McQuade A, Kolahdouzan M, Echeverria K, Claes C, (2019). Development of a Chimeric Model to Study and Manipulate Human Microglia *In Vivo*. Neuron 103, 1016–1033. 31375314 10.1016/j.neuron.2019.07.002 PMC7138101

[R19] Hollingworth P, Harold D, Sims R, Gerrish A, Lambert JC, Carrasquillo MM, Abraham R, Hamshere ML, Pahwa JS, Moskvina V, ; Alzheimer’s Disease Neuroimaging Initiative; CHARGE consortium; EADI1 consortium (2011). Common variants at ABCA7, MS4A6A/MS4A4E, EPHA1, CD33andCD2APareassociatedwithAlzheimer’sdisease. Nat.Genet.43, 429–435. 21460840 10.1038/ng.803 PMC3084173

[R20] Holtman IR, Raj DD, Miller JA, Schaafsma W, Yin Z, Brouwer N, Wes PD, Mö ller T., Orre M, Kamphuis W, (2015). Induction of a common microglia gene expression signature by aging and neurodegenerative conditions: a co-expression meta-analysis. Acta Neuropathol. Commun 3,31. 26001565 10.1186/s40478-015-0203-5 PMC4489356

[R21] Huang KL, Marcora E, Pimenova AA, Di Narzo AF, Kapoor M, Jin SC, Harari O, Bertelsen S, Fairfax BP, Czajkowski J, ; International Genomics of Alzheimer’s Project; Alzheimer’s Disease Neuroimaging Initiative (2017). A common haplotype lowers PU.1 expression in myeloid cells and delays onset of Alzheimer’s disease. Nat. Neurosci. 20, 1052–1061. 28628103 10.1038/nn.4587 PMC5759334

[R22] Jä kel S, Agirre E, Mendanha Falcao A., van Bruggen D, Lee KW, Knue-sel I, Malhotra D, Ffrench-Constant C, Williams A., and Castelo-Branco G (2019). Altered human oligodendrocyte heterogeneity in multiple sclerosis. Nature 566, 543–547. 30747918 10.1038/s41586-019-0903-2 PMC6544546

[R23] Jansen IE, Savage JE, Watanabe K, Bryois J, Williams DM, Steinberg S, Sealock J, Karlsson IK, Hä gg S, Athanasiu L, (2019). Genome-wide meta-analysis identifies new loci and functional pathways influencing Alzheimer’s disease risk. Nat. Genet. 51, 404–413. 30617256 10.1038/s41588-018-0311-9 PMC6836675

[R24] Keren-Shaul H, Spinrad A, Weiner A, Matcovitch-Natan O, Dvir-Sztern-feld R, Ulland TK, David E, Baruch K, Lara-Astaiso D, Toth B, (2017). A Unique Microglia Type Associated with Restricting Development of Alzheimer’s Disease. Cell 169, 1276–1290. 28602351 10.1016/j.cell.2017.05.018

[R25] Kim K, Shim D, Lee JS, Zaitsev K, Williams JW, Kim KW, Jang MY, Seok Jang H, Yun TJ, Lee SH, (2018). Transcriptome Analysis Reveals Nonfoamy Rather Than Foamy Plaque Macrophages Are Proinflammatory in Atherosclerotic Murine Models. Circ. Res. 123, 1127–1142. 30359200 10.1161/CIRCRESAHA.118.312804 PMC6945121

[R26] Krasemann S, Madore C, Cialic R, Baufeld C, Calcagno N, El Fatimy R, Beckers L, O’Loughlin E, Xu Y, Fanek Z, (2017). The TREM2-APOE Pathway Drives the Transcriptional Phenotype of Dysfunctional Microglia in Neurodegenerative Diseases. Immunity 47, 566–581. 28930663 10.1016/j.immuni.2017.08.008 PMC5719893

[R27] Kunkle BW, Grenier-Boley B, Sims R, Bis JC, Damotte V, Naj AC, Boland A, Vronskaya M, van der Lee SJ, Amlie-Wolf A, ; Alzheimer Disease Genetics Consortium (ADGC); European Alzheimer’s Disease Initiative (EADI); Cohorts for Heart and Aging Research in Genomic Epidemiology Consortium (CHARGE); Genetic and Environmental Risk in AD/Defining Genetic, Polygenic and Environmental Risk for Alzheimer’s Disease Consortium (GERAD/PERADES) (2019). Genetic meta-analysis of diagnosed Alzheimer’s disease identifies new risk loci and implicates Ab, tau, immunity and lipid processing. Nat. Genet. 51, 414–430. 30820047 10.1038/s41588-019-0358-2 PMC6463297

[R28] Lambert JC, Ibrahim-Verbaas CA, Harold D, Naj AC, Sims R, Bellenguez C, DeStafano AL, Bis JC, Beecham GW, Grenier-Boley B, ; European Alzheimer’s Disease Initiative (EADI); Genetic and Environmental Risk in Alzheimer’s Disease; Alzheimer’s Disease Genetic Consortium; Cohorts for Heart and Aging Research in Genomic Epidemiology (2013). Meta-analysis of 74,046 individuals identifies 11 new susceptibility loci for Alzheimer’s disease. Nat. Genet. 45, 1452–1458. 24162737 10.1038/ng.2802 PMC3896259

[R29] Lavin Y, Winter D, Blecher-Gonen R, David E, Keren-Shaul H, Merad M, Jung S, and Amit I (2014). Tissue-resident macrophage enhancer landscapes are shaped by the local microenvironment. Cell 159, 1312–1326. 25480296 10.1016/j.cell.2014.11.018 PMC4437213

[R30] Marioni RE, Harris SE, Zhang Q, McRae AF, Hagenaars SP, Hill WD, Davies G, Ritchie CW, Gale CR, Starr JM, (2018). GWAS on family history of Alzheimer’s disease. Transl. Psychiatry 8,99. 29777097 10.1038/s41398-018-0150-6 PMC5959890

[R31] Masuda T, Sankowski R, Staszewski O, Bö ttcher C, Amann L, Scheiwe Sagar C, Nessler S., Kunz P., van Loo G, (2019). Spatial and temporal heterogeneity of mouse and human microglia at single-cell resolution. Nature 566, 388–392. 30760929 10.1038/s41586-019-0924-x

[R32] Mathys H, Davila-Velderrain J, Peng Z, Gao F, Mohammadi S, Young JZ, Menon M, He L, Abdurrob F, Jiang X, (2019). Single-cell transcriptomic analysis of Alzheimer’s disease. Nature 570, 332–337. 31042697 10.1038/s41586-019-1195-2 PMC6865822

[R33] Mirra SS, Heyman A, McKeel D, Sumi SM, Crain BJ, Brownlee LM, Vogel FS, Hughes JP, van Belle G, and Berg L (1991). The Consortium to Establish a Registry for Alzheimer’s Disease (CERAD). Part II. Standardization of the neuropathologic assessment of Alzheimer’s disease. Neurology 41, 479–486. 2011243 10.1212/wnl.41.4.479

[R34] Naj AC, Jun G, Beecham GW, Wang LS, Vardarajan BN, Buros J, Gallins PJ, Buxbaum JD, Jarvik GP, Crane PK, (2011). Common variants at MS4A4/MS4A6E, CD2AP, CD33 and EPHA1 are associated with late-onset Alzheimer’s disease. Nat. Genet. 43, 436–441. 21460841 10.1038/ng.801 PMC3090745

[R35] Novikova G, Kapoor M, Tcw J, Abud EM, Efthymiou AG, Cheng H, Fullard JF, Bendl J, Roussos P, Poon WW, (2019). Integration of Alzheimer’s disease genetics and myeloid cell genomics identifies novel causal variants, regulatory elements, genes and pathways. bioRxiv. 10.1038/s41467-021-21823-y PMC7955030 33712570

[R36] Nugent AA, Lin K, van Lengerich B, Lianoglou S, Przybyla L, Davis SS, Llapashtica C, Wang J, Kim DJ, Xia D, (2020). TREM2 Regulates Microglial Cholesterol Metabolism upon Chronic Phagocytic Challenge. Neuron 105, 837–854. 31902528 10.1016/j.neuron.2019.12.007

[R37] Olah M, Patrick E, Villani AC, Xu J, White CC, Ryan KJ, Piehowski P, Kapasi A, Nejad P, Cimpean M, (2018). A transcriptomic atlas of aged human microglia. Nat. Commun. 9, 539. 29416036 10.1038/s41467-018-02926-5 PMC5803269

[R38] Orre M, Kamphuis W, Osborn LM, Jansen AHP, Kooijman L, Bossers K, and Hol EM (2014). Isolation of glia from Alzheimer’s mice reveals inflammation and dysfunction. Neurobiol. Aging 35, 2746–2760. 25002035 10.1016/j.neurobiolaging.2014.06.004

[R39] Poliani PL, Wang Y, Fontana E, Robinette ML, Yamanishi Y, Gilfillan S, and Colonna M (2015). TREM2 sustains microglial expansion during aging and response to demyelination. J. Clin. Invest. 125, 2161–2170. 25893602 10.1172/JCI77983 PMC4463196

[R40] Ramanan VK, Risacher SL, Nho K, Kim S, Shen L, McDonald BC, Yoder KK, Hutchins GD, West JD, Tallman EF, ; Alzheimer’s Disease Neuroimaging Initiative (ADNI) (2015). GWAS of longitudinal amyloid accumulation on 18F-florbetapir PET in Alzheimer’s disease implicates microglial activation gene IL1RAP. Brain 138, 3076–3088. 26268530 10.1093/brain/awv231 PMC4671479

[R41] Rathore N, Ramani SR, Pantua H, Payandeh J, Bhangale T, Wuster A, Kapoor M, Sun Y, Kapadia SB, Gonzalez L, (2018). Paired Immunoglobulin-like Type 2 Receptor Alpha G78R variant alters ligand binding and confers protection to Alzheimer’s disease. PLoS Genet 14, e1007427. 30388101 10.1371/journal.pgen.1007427 PMC6235402

[R42] Sims R, van der Lee SJ, Naj AC, Bellenguez C, Badarinarayan N, Ja-kobsdottir J, Kunkle BW, Boland A, Raybould R, Bis JC, ; ARUK Consortium; GERAD/PERADES, CHARGE, ADGC, EADI (2017). Rare coding variants in PLCG2, ABI3, and TREM2 implicate microglial-mediated innate immunity in Alzheimer’s disease. Nat. Genet. 49, 1373–1384. 28714976 10.1038/ng.3916 PMC5669039

[R43] Srinivasan K, Friedman BA, Larson JL, Lauffer BE, Goldstein LD, Appling LL, Borneo J, Poon C, Ho T, Cai F, (2016). Untangling the brain’s neuroinflammatory and neurodegenerative transcriptional responses. Nat. Commun. 7, 11295. 27097852 10.1038/ncomms11295 PMC4844685

[R44] Streit WJ, Braak H, Xue QS, and Bechmann I (2009). Dystrophic (senescent) rather than activated microglial cells are associated with tau pathology and likely precede neurodegeneration in Alzheimer’s disease. Acta Neuropathol. 118, 475–485. 19513731 10.1007/s00401-009-0556-6 PMC2737117

[R45] Valdé s Herná ndez MDC, Reid S, Mikhael S, and Pernet C; Alzheimer’s Disease Neuroimaging Initiative (2018). Do 2-year changes in superior frontal gyrus and global brain atrophy affect cognition? Alzheimers Dement. (Amst.) 10, 706–716. 30511008 10.1016/j.dadm.2018.07.010 PMC6258225

[R46] Wang Y, Cella M, Mallinson K, Ulrich JD, Young KL, Robinette ML, Gilfillan S, Krishnan GM, Sudhakar S, Zinselmeyer BH, (2015). TREM2 lipid sensing sustains the microglial response in an Alzheimer’s disease model. Cell 160, 1061–1071. 25728668 10.1016/j.cell.2015.01.049 PMC4477963

[R47] Zhang Y, Sloan SA, Clarke LE, Caneda C, Plaza CA, Blumenthal PD, Vogel H, Steinberg GK, Edwards MS, Li G, (2016). Purification and Characterization of Progenitor and Mature Human Astrocytes Reveals Transcriptional and Functional Differences with Mouse. Neuron 89, 37–53. 26687838 10.1016/j.neuron.2015.11.013 PMC4707064

