# Supplementary material for: Alzheimer’s Patient Microglia Exhibit Enhanced Aging and Unique Transcriptional Activation
Source: Cell Rep. Author manuscript; Available in PMC 2020 Aug 12. (PMC7422733; doi:10.1016/j.celrep.2020.107843)
Supplement: 1 [file NIHMS1608497-supplement-1.pdf]

Cell Reports, Volume *31*

Supplemental Information

Alzheimer's Patient Microglia Exhibit Enhanced Aging and Unique Transcriptional Activation

Karpagam Srinivasan, Brad A. Friedman, Ainhoa Etxeberria, Melanie A. Huntley, Marcel P. van der Brug, Oded Foreman, Jonathan S. Paw, Zora Modrusan, Thomas G. Beach, Geidy E. Serrano, and David V. Hansen

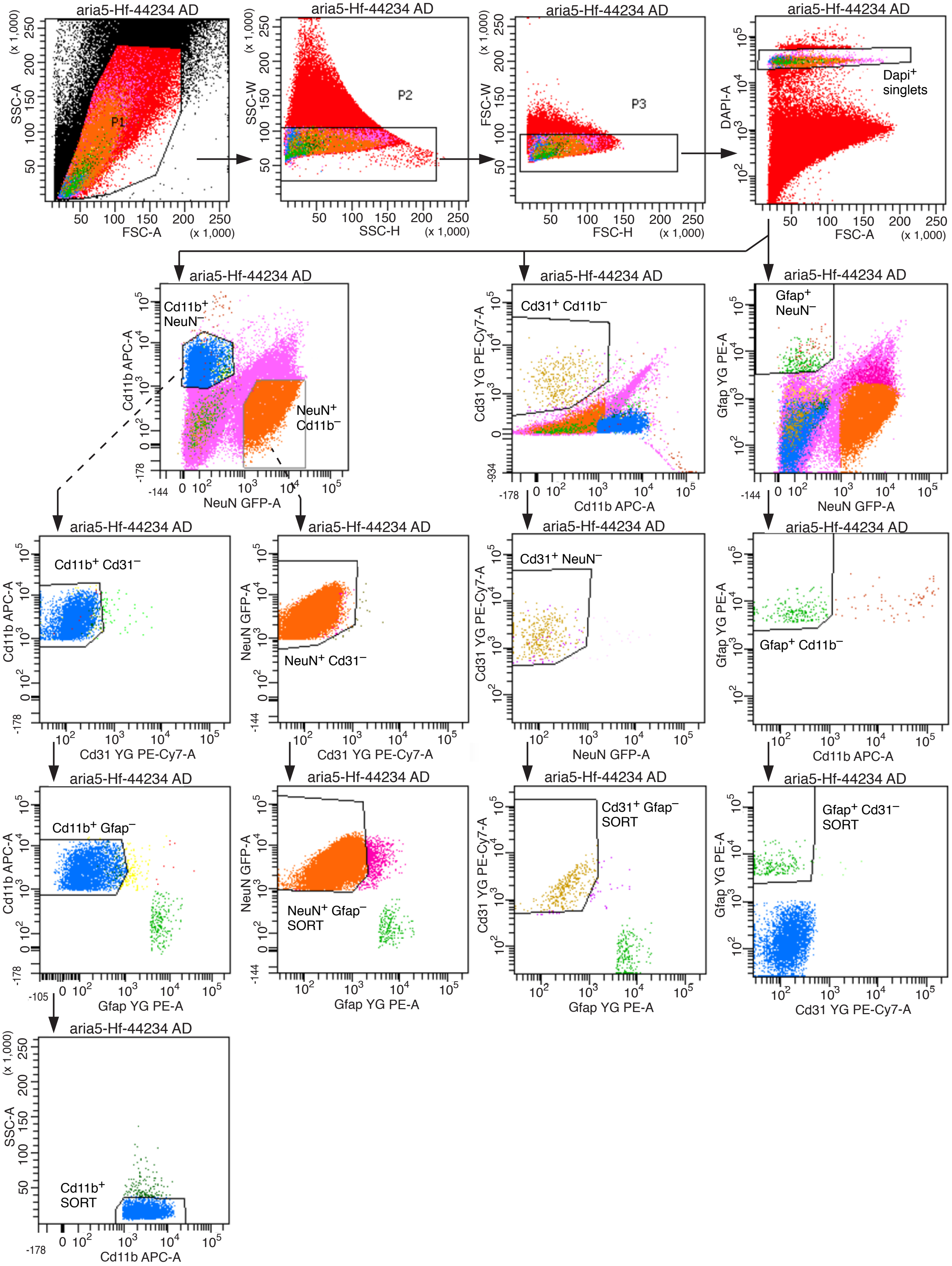
aria5-Hf-44234 AD
SSC-A (x 1,000)
FSC-A (x 1,000)
P1
SSC-W (x 1,000)
SSC-H (x 1,000)
P2
FSC-W (x 1,000)
FSC-H (x 1,000)
P3
DAPI-A
Dapi+ singlets
Cd11b+ NeuN−
NeuN+ Cd11b−
Cd11b APC-A
NeuN GFP-A
Cd31+ Cd11b−
Cd31 YG PE-Cy7-A
Gfap+ NeuN−
Gfap YG PE-A
Cd11b+ Cd31−
NeuN+ Cd31−
Cd31+ NeuN−
Gfap+ Cd11b−
Cd11b+ Gfap−
NeuN+ Gfap− SORT
Cd31+ Gfap− SORT
Gfap+ Cd31− SORT
Cd11b+ SORT

Figure S1. Example FACS plots showing isolation of four cell type populations from one AD sample. Related to Figure 1A.

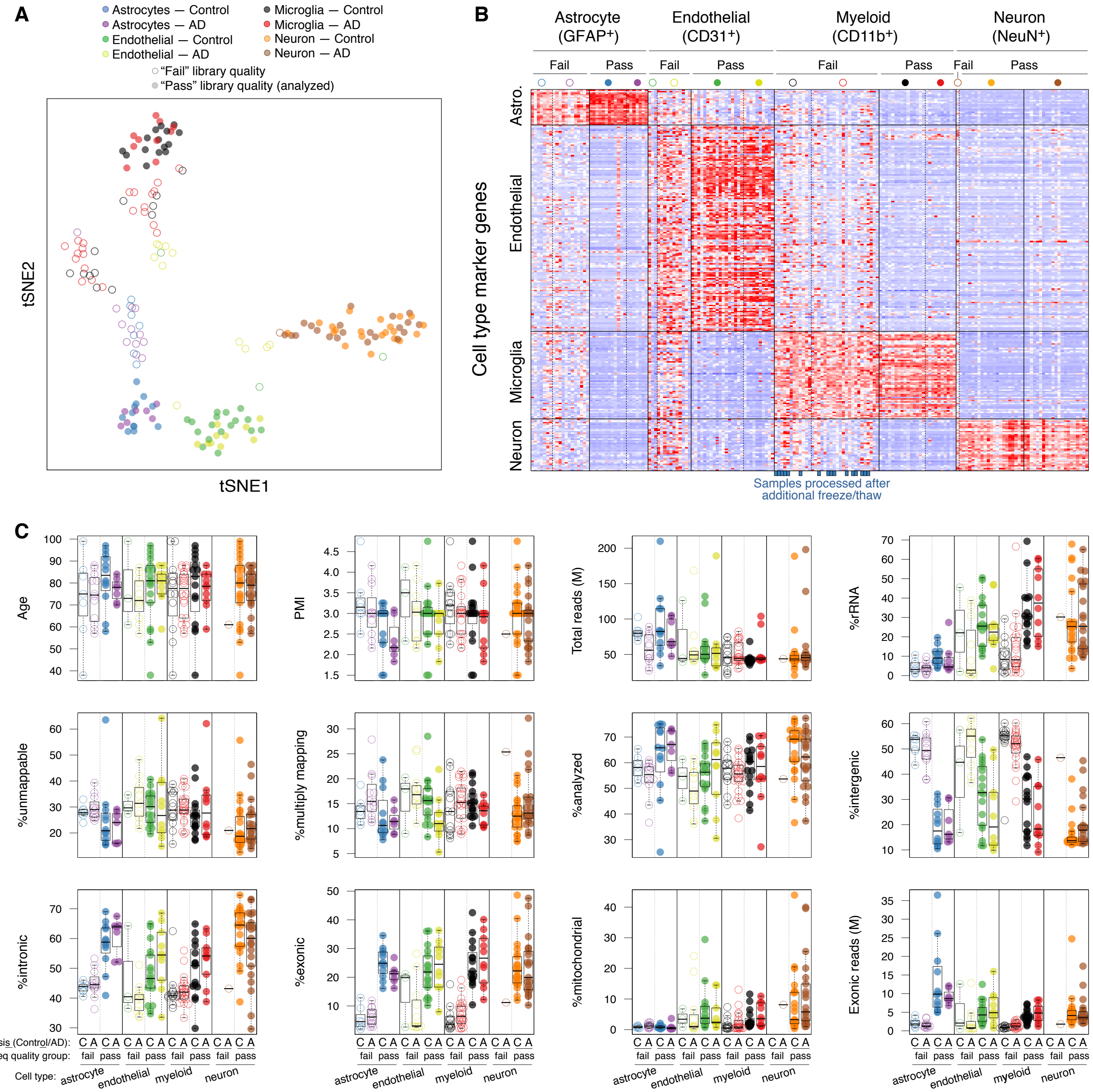

Figure S2. Quality control of SFG RNA-Seq profiles. Related to Figure 1B-1D.
(A) tSNE analysis of passing and failing RNA-Seq profiles. Note that separation of cell types degrades in "Fail" samples.
(B) Heatmap of all samples. Solid vertical lines separate cell types, and Pass/Fail within cell types. Dashed lines separate Control and AD samples. Libraries from 16 samples which were processed after repeated freeze/thaw are indicated below. "Fail" samples often displayed less specific expression of cell type markers.
(C) Subject, sample and library attributes separated by cell type, quality group (Pass/Fail) and diagnosis. Age and PMI in particular do not appear to show strong differences between Pass and Fail samples, although some RNA-Seq library statistics, such as %exonic and %intergenic, do appear quite different. Denominators for percentages as follows. %rRNA: total reads; %unmappable, %multiply mapping, %uniquely mapping: "processed" reads (total reads

with rRNA, low quality, and adapter contamination removed); %intergenic, %intronic, %exonic, %mitochondrial: total uniquely mapping reads.

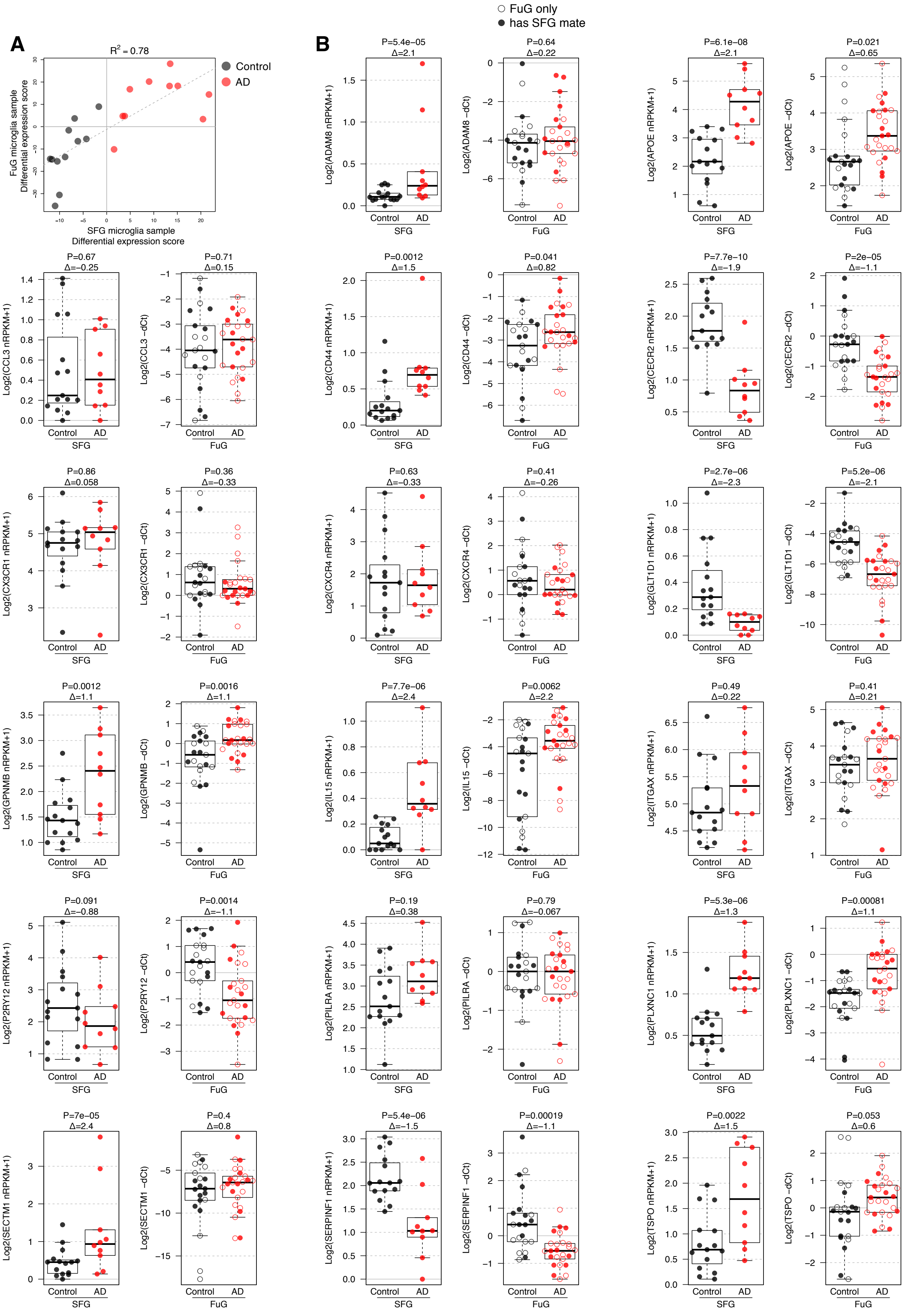
FuG only
has SFG mate
A
B
R² = 0.78
Control
AD
FuG microglia sample
Differential expression score
SFG microglia sample
Differential expression score
P=5.4e−05 Δ=2.1
Log2(ADAM8 nRPKM+1)
P=0.64 Δ=0.22
Log2(ADAM8 −dCt)
P=6.1e−08 Δ=2.1
Log2(APOE nRPKM+1)
P=0.021 Δ=0.65
Log2(APOE −dCt)
P=0.67 Δ=−0.25
Log2(CCL3 nRPKM+1)
P=0.71 Δ=0.15
Log2(CCL3 −dCt)
P=0.0012 Δ=1.5
Log2(CD44 nRPKM+1)
P=0.041 Δ=0.82
Log2(CD44 −dCt)
P=7.7e−10 Δ=−1.9
Log2(CECR2 nRPKM+1)
P=2e−05 Δ=−1.1
Log2(CECR2 −dCt)
P=0.86 Δ=0.058
Log2(CX3CR1 nRPKM+1)
P=0.36 Δ=−0.33
Log2(CX3CR1 −dCt)
P=0.63 Δ=−0.33
Log2(CXCR4 nRPKM+1)
P=0.41 Δ=−0.26
Log2(CXCR4 −dCt)
P=2.7e−06 Δ=−2.3
Log2(GLT1D1 nRPKM+1)
P=5.2e−06 Δ=−2.1
Log2(GLT1D1 −dCt)
P=0.0012 Δ=1.1
Log2(GPNMB nRPKM+1)
P=0.0016 Δ=1.1
Log2(GPNMB −dCt)
P=7.7e−06 Δ=2.4
Log2(IL15 nRPKM+1)
P=0.0062 Δ=2.2
Log2(IL15 −dCt)
P=0.49 Δ=0.22
Log2(ITGAX nRPKM+1)
P=0.41 Δ=0.21
Log2(ITGAX −dCt)
P=0.091 Δ=−0.88
Log2(P2RY12 nRPKM+1)
P=0.0014 Δ=−1.1
Log2(P2RY12 −dCt)
P=0.19 Δ=0.38
Log2(PILRA nRPKM+1)
P=0.79 Δ=−0.067
Log2(PILRA −dCt)
P=5.3e−06 Δ=1.3
Log2(PLXNC1 nRPKM+1)
P=0.00081 Δ=1.1
Log2(PLXNC1 −dCt)
P=7e−05 Δ=2.4
Log2(SECTM1 nRPKM+1)
P=0.4 Δ=0.8
Log2(SECTM1 −dCt)
P=5.4e−06 Δ=−1.5
Log2(SERPINF1 nRPKM+1)
P=0.00019 Δ=−1.1
Log2(SERPINF1 −dCt)
P=0.0022 Δ=1.5
Log2(TSPO nRPKM+1)
P=0.053 Δ=0.6
Log2(TSPO −dCt)
Control AD
SFG
FuG

Figure S3. Differentially expressed (DE) genes in AD microglia from SFG RNA-Seq data are largely reproduced in FuG qPCR data. Related to Figures 3A-3C.
(A) DE scores were calculated for sorted microglia from SFG and FuG of the same subjects, with higher scores indicating increased degree of differential expression for the genes identified as DE by RNA-Seq in the SFG samples and present in the qPCR panel. Each point represents one subject for which passing SFG RNA-Seq and FuG qPCR profiles were available, with coordinates giving the DE scores (Methods) for corresponding SFG and FuG profiles.
(B) Selected examples of gene expression measurements in SFG microglia by RNA-Seq and FuG microglia by qPCR. Depicted are 5 of 16 tested HAM-Up genes (*ADAM8*, *APOE*, *IL15*, *PLXNC1*, and *SECTM1*), 3 of 6 tested HAM-Down genes (*CECR2*, *GLT1D1*, *SERPINF1*), five DAM genes (*APOE*, *CCL3*, *CXCR4*, *GPNMB*, *ITGAX*), two "homeostatic" or "resting" microglia genes (*CX3CR1*, *P2RY12*) and other genes of interest (*CD44*, *PILRA*, *TSPO*). Whereas Figure 3B used adjusted P-values for both datasets, this figure panel lists unadjusted P-values for consideration of individual genes. Thus, *CD44*, *GPNMB*, and *TSPO* appear upregulated in SFG AD microglia in this figure, with unadjusted P-values ≤ 0.05. The lack of replication in FuG microglia for *ADAM8* and *SECTM1* being upregulated in AD could be because the DE signal for these genes in SFG microglia was driven primarily by two outlier samples in the AD group. (See Data S2, columns EK-GH, for the entire qPCR panel's gene expression values across all FuG microglia samples.)

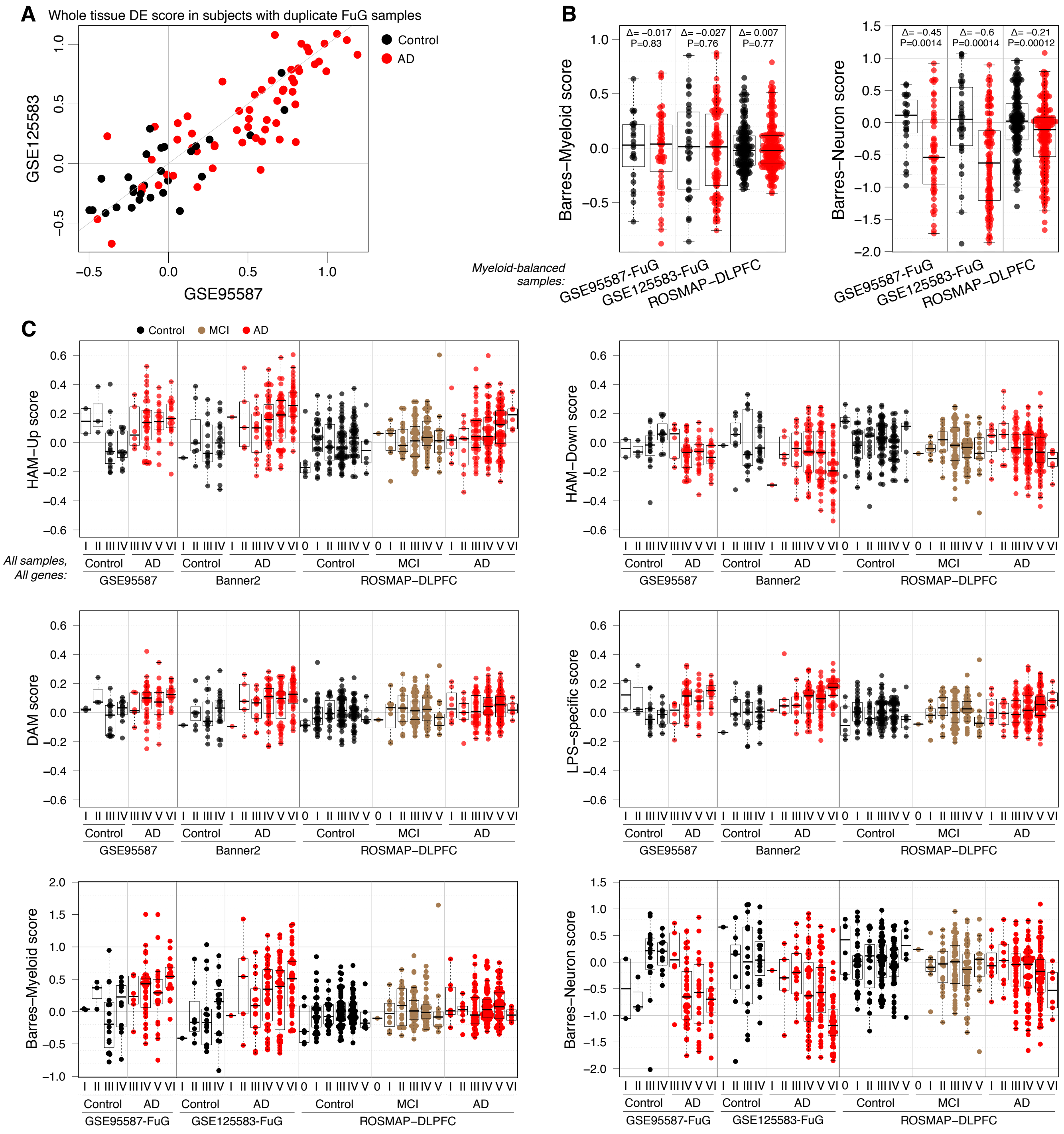

Figure S4. Whole tissue RNA analyses validate HAM signature in late-stage AD. Related to Figure 3D.
(A) Duplicated FuG samples show consistent DE. 89 samples were duplicated in GSE95587 and GSE125583, in the sense that they came from different tissue blocks of the same fusiform gyrus. For each of these, a sample-wise DE score was calculated separately in the two datasets using common DE genes. Plot shows that the DE scores are highly correlated, indicating that the expression signature of a small piece of tissue reflects the entire brain region.
(B) Myeloid-balancing results in similar distributions of myeloid scores but still a strong depletion of neuron gene expression in whole AD brain tissue RNA. Plot shows gene set scores of indicated gene sets in individual whole tissue RNA samples from three different cohorts, similar to Fig. 3D but for different gene sets. Also, neuronal genes were

not removed (that would not be meaningful in this context since none of the Barres-Myeloid and all of the Barres-Neuron genes are neuronal).

(C) Gene set changes are observed in whole AD tissues at later Braak stages, with HAM-Up scores being larger than scores for mouse-derived DAM or LPS-specific gene sets. Plots are similar to Fig. 3D but include all samples and genes, without myeloid balancing or removing neuronal genes, and with samples stratified by Braak stage.

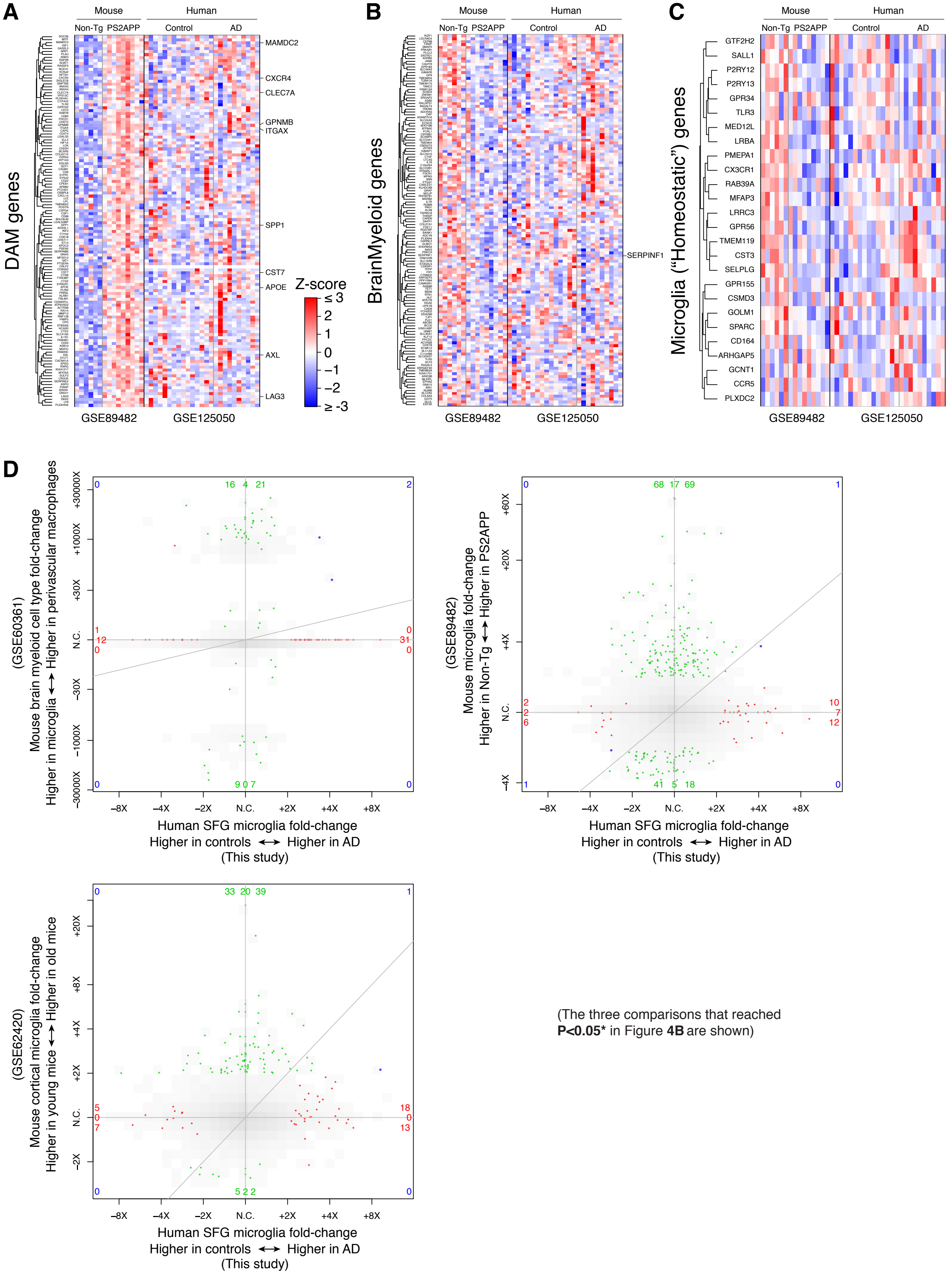

A
Mouse
Human
Non-Tg
PS2APP
Control
AD
DAM genes
MAMDC2
CXCR4
CLEC7A
GPNMB
ITGAX
SPP1
CST7
APOE
AXL
LAG3
GSE89482
GSE125050
Z-score
≤ 3
2
1
0
−1
−2
≥ -3
B
BrainMyeloid genes
SERPINF1
C
Microglia ("Homeostatic") genes
GTF2H2
SALL1
P2RY12
P2RY13
GPR34
TLR3
MED12L
LRBA
PMEPA1
CX3CR1
RAB39A
MFAP3
LRRC3
GPR56
TMEM119
CST3
SELPLG
GPR155
CSMD3
GOLM1
SPARC
CD164
ARHGAP5
GCNT1
CCR5
PLXDC2
D
(GSE60361)
Mouse brain myeloid cell type fold-change
Higher in microglia ⟷ Higher in perivascular macrophages
Human SFG microglia fold-change
Higher in controls ⟷ Higher in AD
(This study)
(GSE89482)
Mouse microglia fold-change
Higher in Non-Tg ⟷ Higher in PS2APP
(GSE62420)
Mouse cortical microglia fold-change
Higher in young mice ⟷ Higher in old mice
(The three comparisons that reached
P<0.05* in Figure 4B are shown)

Figure S5. Differential expression patterns observed in mouse microglia are not conserved in human AD microglia. Related to Figure 4.
(A-C) Heatmaps of DAM (Neurodegeneration-related) gene modules (A), BrainMyeloid gene modules (B), and "homeostatic" Microglia gene module expression in $CD11b^+$ microglia/myeloid cells sorted from the PS2APP mouse model of β-amyloidosis versus non-transgenic mice (GSE89482), or sorted from frozen human SFG of AD versus control subjects (this study, GSE125050). Gene modules were defined in previous study (Friedman et al., 2018). The microglial responses commonly observed in PS2APP and other mouse neurodegeneration models—namely, the induction of DAM genes and the downregulation of BrainMyeloid/homeostatic microglia genes—are largely absent in human AD microglia. Overall sample-wise scores for these and other mouse-derived gene modules in human AD vs. control microglia are shown in Figure 4A.
(D) "4-way" plots for the three mouse datasets for which the change in DE scores shown in Figure 4B was significant. Each point represents one gene from the mouse DE profile, with the gene's fold-change in the respective mouse dataset plotted on the *y*-axis and its fold-change in human AD vs. control SFG microglia plotted on the *x*-axis. Very few genes are significantly altered in both datasets, and overall correlations between up- and down-regulation of DE genes between datasets are not obvious.

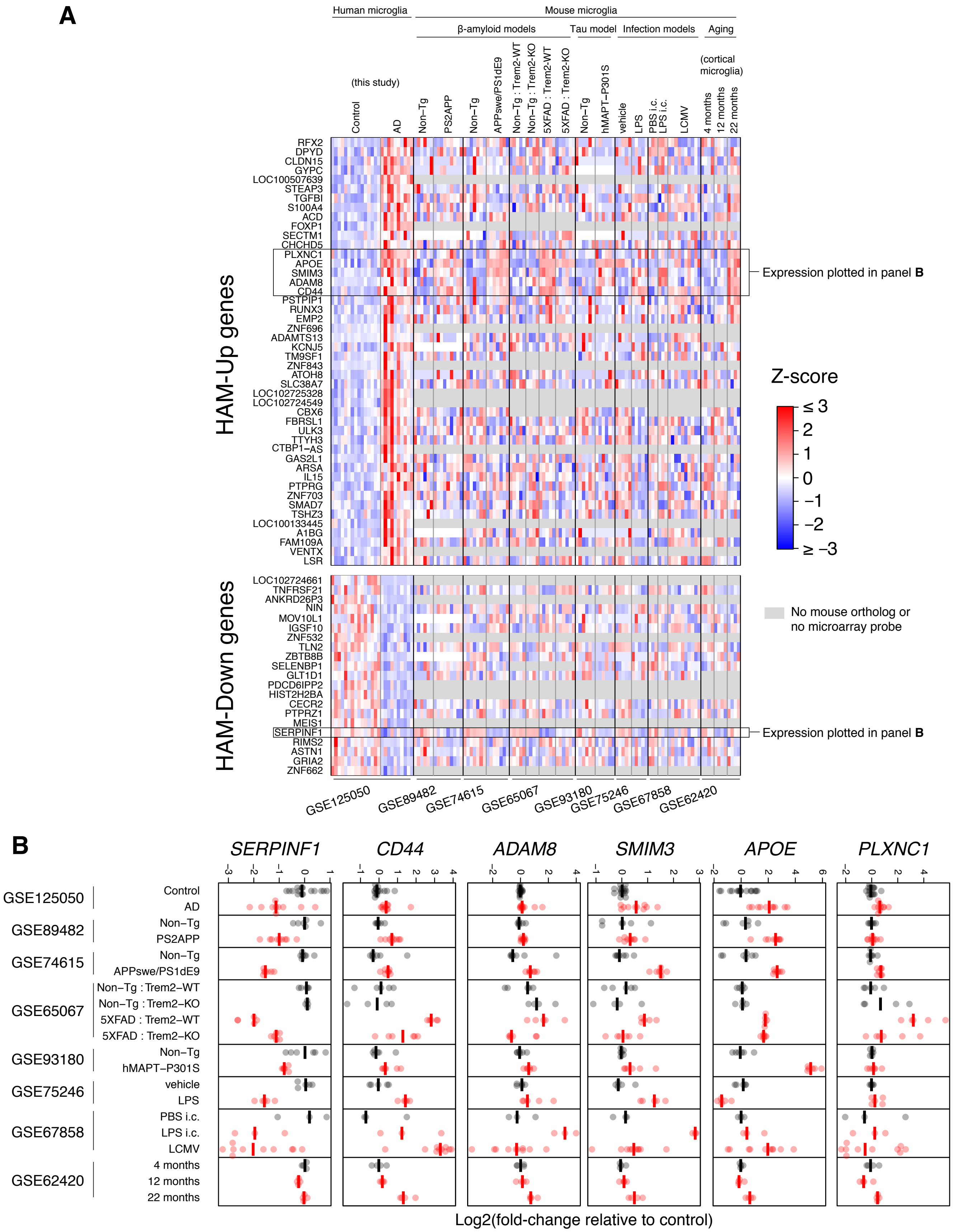

Figure S6. Few of the transcriptional changes observed in human Alzheimer's microglia are observed in mouse models of neurodegeneration or other microglia-activating stimuli. Related to Figure 4.

(A) Heatmaps showing the expression of human AD microglia (HAM) DE genes (and CD44, see Methods) in mouse microglia datasets from models of β-amyloidosis, tauopathy model, infection, and aging.

(B) Expression of selected HAM profile genes (the few which exhibit consistent changes in mouse datasets) in individual samples in mouse datasets. Expression values are normalized to average expression in the control group within each study.

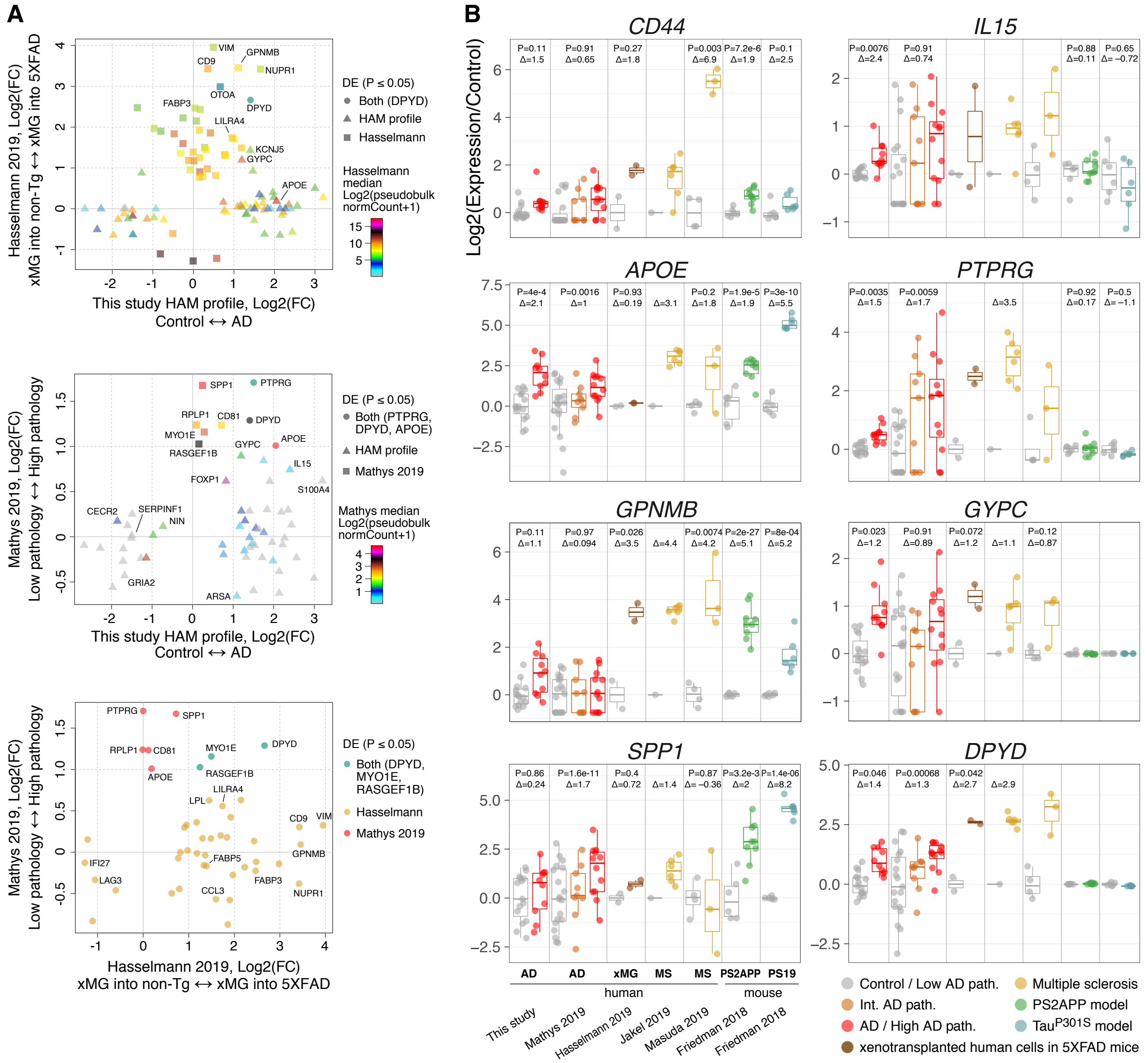

Figure S7. Comparisons of differential expression for individual genes in human microglia/myeloid cell RNA-Seq datasets related to neurodegenerative diseases and in mouse models of amyloid or tau pathologies. Related to Figure 7.

(A) "4-way" plots comparing differential expression in each pairing of three AD-related human microglia studies. Points are genes significant in at least one of the two indicated studies, with axes showing corresponding log2-fold-changes. Shape of the points (first two plots) or color of the points (last plot) indicate whether the differential expression of the indicated gene was statistically significant in one or both studies. In the first two plots, the points are colored by the pseudobulk expression in the single-cell/nucleus studies. Note that in particular, in Mathys 2019, many of the HAM-Up genes (on the right side) and HAM-Down genes (on the left side) were gray, indicating low detection rates in that study.

(B) Control-centered expression of example genes with elevated human microglial expression in various neurodegenerative settings. Organization of studies is identical to Figure 7. Left column genes are genes that also show some elevation in mouse models. Right column are examples of HAM genes that do not change (*PTPRG*, *IL15*) or are barely expressed (*DPYD*, *GYPC*) in mouse datasets. Adjusted *P*-values and Δ (log2-fold-changes) come from corresponding differential expression analyses described in the methods. The apparent discrepancy in Δ in some datasets (e.g., *SPP1* in the mouse datasets) relative to the data plotted is due to the plotted values being stabilized via

log2(x+1) transformation. *P*-values are omitted from the Jakel 2019 dataset (GSE118257) since only one control sample had enough (≥ 10) cells in the relevant tSNE clusters for pseudobulk analysis (see Data S4 panel 3). Other missing statistics are genes that were omitted due to "low expression" in the differential expression analysis. See Methods for more information on differential expression analyses.

Gene Set	Mouse genes	Human orthologs	Analyzed[a]	Trending up[b]	Trending down[b]	Differentially expressed ($P \leq 0.05$)[c]	Notable strongly trending genes
Interferon-Related	31	27	23	7	16		
Neurodegeneration -Related/DAM	134	126	112	55	56	*APOE* (FC=4.1, P=0.0004)	*APOC1* (FC=3.0, P=0.056), *GPNMB* (FC=2.2, P=0.11)
Microglia	27	26	25	10	15		
Macrophage	15	14	10	5	4	*TGFBI* (FC=3.4, P=0.0042)	
Monocyte/ Neutrophil	34	28	24	17	7		
Proliferation	82	81	67	27	40		
LPS-Related	81	76	69	42	26	*ADAM8* (FC=4.4, P=0.025)	*TSPO* (FC=2.8, P=0.14), *CD44* (FC=2.8, P=0.11)
BrainMyeloid	136	132	126	60	65	*SERPINF1* (FC=-2.8, P=0.006)	
LPS-specific	57	51	42	22	20		

Table S1. Analysis of differential expression in human AD myeloid cells for orthologs of mouse-derived gene sets. Related to Figure 4A.

[a] A few of the human orthologs were not analyzed either because the genes were either low expressed or because the calculated fold-changes and P-values were determined by Cook's distance to be driven by outliers (see Methods). The number of "Analyzed" genes in each set is exactly equal to the number of genes in the next three columns: Trending up, Trending down, and Differentially Expressed.

[b] The terms "Trending up" and "Trending down" do not imply strong trends. The terms refer simply to whether the fold-change (FC) values were positive or negative (respectively) for all genes that were not differentially expressed in the DESeq2 analysis (i.e., all genes with P-values > 0.05).

[c] P-values shown are adjusted for genome-wide multiple testing (see Methods).
