# Supplementary material for: Alzheimer’s Patient Microglia Exhibit Enhanced Aging and Unique Transcriptional Activation
Source: Cell Rep. Author manuscript; Available in PMC 2020 Aug 12. (PMC7422733; doi:10.1016/j.celrep.2020.107843)
Supplement: 5 [file NIHMS1608497-supplement-5.pdf]

Analyses of Non-disease Covariates, and Analyses of Human Myeloid Single Nucleus and Single Cell RNA-Seq Datasets

Contents of this file

- Panel 1: Analyses of non-disease covariates (post-mortem interval, sex, aging)
- Panel 2: Analyses of Alzheimer’s patient snRNA-Seq and xenotransplanted scRNA-Seq datasets
- Panel 3: Analyses of multiple sclerosis sn/scRNA-Seq datasets

Panel 1. Analyses of non-disease covariates

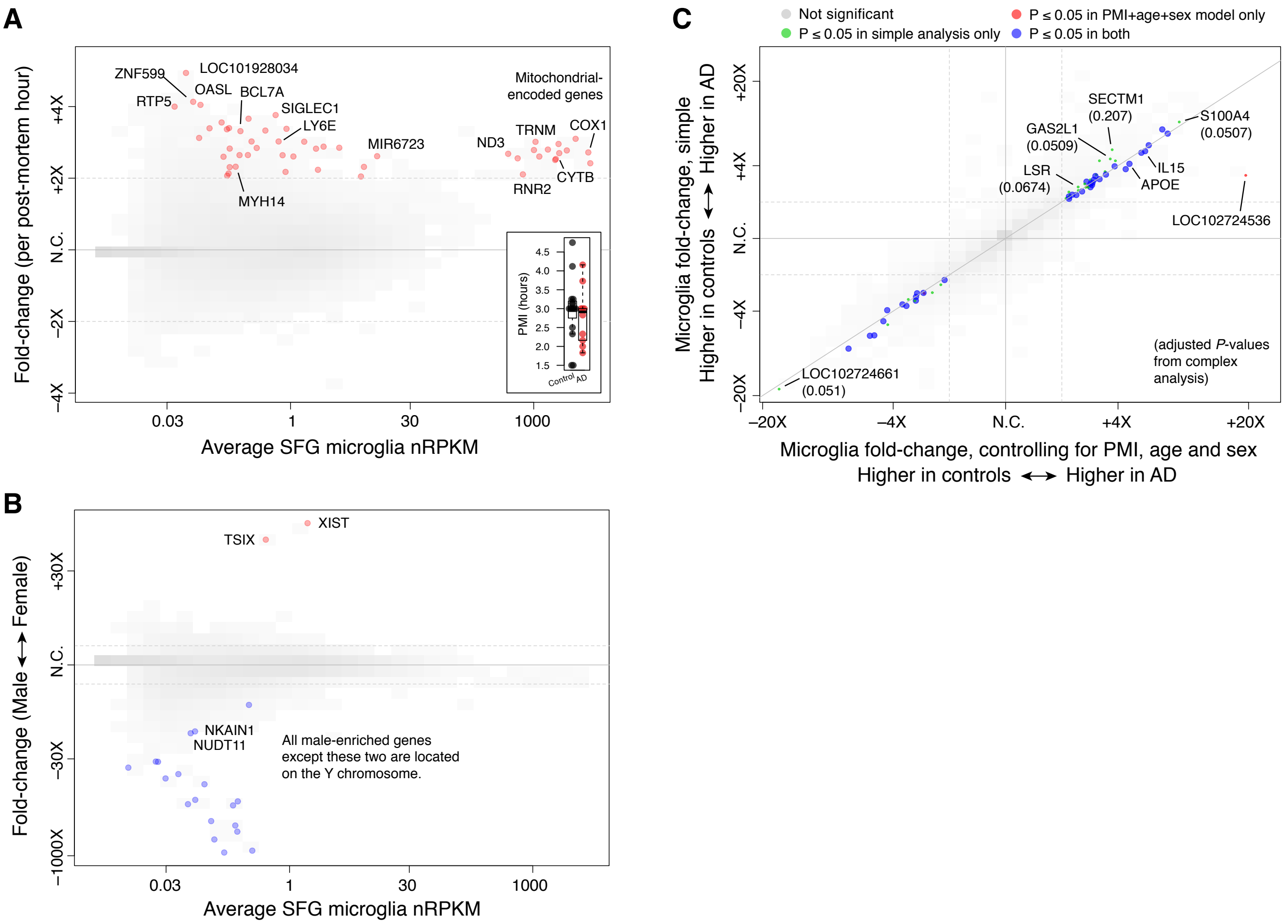

Controlling for sex, age, and PMI has limited effect on SFG microglia differential expression (DE) results.
(A) M-A plot showing genes differentially expressed with post-mortem interval (PMI). The model ~ PMI was used with DESeq2 to analyze the myeloid samples. x-axis, average gene expression; y-axis, fold-change per hour PMI. Significantly DE genes (fold-change ≥ 2, adjusted P ≤ 0.05) shown in red. All the genes in the right "blob" are located on the mitochondrial chromosome. None of the red genes were DE in main diagnosis analysis. Inset, distribution of PMI for microglia samples, repeated from Figure S2.
(B) Similar to (a), but for male versus female differential expression in microglia. Female-enriched genes (red) were limited to *XIST* and *TSIX*, as expected, and, aside from the two genes indicated, male-enriched genes (blue) were limited to Y-chromosome genes.
(C) "4-way" plot comparing complex DE analysis (x-axis), including terms for age, PMI and sex, to simple two-group (AD versus control) analysis (y-axis). For the "complex" analysis the model ~diagnosis+PMI+sex+age was analyzed with DESeq2 and the diagnosis term is shown. Fold-changes of DE genes (fold-change ≥ 2, adjusted P ≤ 0.05, Cook's P-value ≥ 0.05) in either analysis are plotted. Most of these genes are near the line y=x, indicating that the fold-changes are highly concordant between the two models. The green-colored genes did not meet the adjusted P ≤ 0.05 threshold in the complex analysis, although many were close (selected adjusted P-values from complex analysis shown in parentheses). In fact, with the exception of *SECTM1* (P=0.207) all of the complex adjusted P-values were less than 0.2, consistent with a slight reduction in power for the more complicated model.

Panel 2. Analyses of Alzheimer’s patient snRNA-Seq and xenotransplanted scRNA-Seq datasets

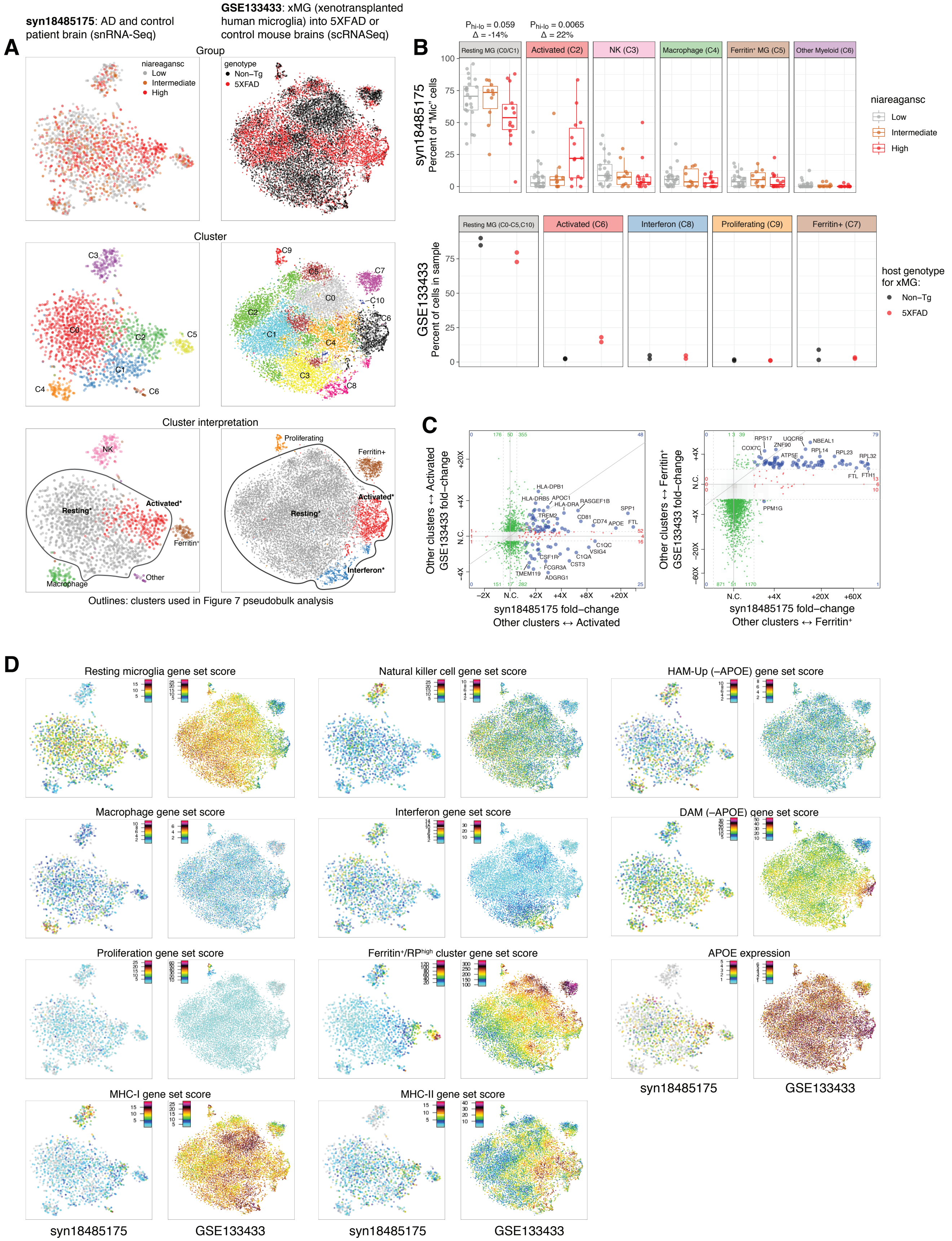

Post-mortem AD tissue microglia and xenotransplanted human microglia (xMG) exhibit similarities and differences in response to AD pathology in human and mouse.

(A) Cells classified by authors as broad.cell.type="mic" in post-mortem snRNA-Seq from AD and control (syn18485175) or all human myeloid cells xenotransplanted into 5XFAD or control (GSE133433) were analyzed by tSNE projection and clustering with Seurat (see Methods). Post-mortem cells were classified into "Pathology Groups" by NIA Reagan score of donor brains, based on a combination of beta-amyloid and tau pathology, and xMG were classified by genotype of host brain (first row). Unbiased Seurat clustering was performed within each study (second row) and interpreted (third row, with justification in following panels).

(B) "Activated" cluster is associated with Alzheimer's pathology in both datasets. Each point represents the percent of cells in a sample, relative to total number of analyzed cells (within "mic" class, syn18485175; entire dataset, GSE133433) in the indicated clusters. Color corresponds to last row of panel a. P values are from t-test comparing percentages of cells in "high" versus "low" pathology groups; Δ are the differences in means of the groups. Missing P values in syn18485175 were not significant, and no statistical tests were performed in GSE133433 due to low number of samples.

(C) "4way" analyses comparing gene enrichment in indicated clusters between the two datasets. Mann-Whitney test was performed to identify markers enriched within each dataset in cells in the indicated clusters relative to cells in other clusters (see Methods). Color indicates whether genes were significantly enriched at adjusted $P \leq 0.05$ and $|\log2(\text{fold-change})| \geq 1.2$ ("Activated") or 1 ("Ferritin+") in one or both datasets. "Activated" cells share in common elevation of MHC-II genes, some DAMs (*SPP1*, *APOE*, *TREM2*) and *FTL*, but differ in homeostatic microglia markers, which are down in xMG Activated cells, but elevated in high-pathology post-mortem cells. "Ferritin+" cells, which were also high for ribosomal protein and electron transport genes, show similar gene expression differences in both datasets, indicating either shared biology or shared artifact.

(D) tSNE plots, as in (a), colored by gene expression of indicated gene sets, or *APOE* gene alone. "Resting Microglia", "Macrophage", "Proliferation", "Interferon" and "DAM (-APOE)" were human orthologs of previously described mouse gene sets3, with "DAM (-APOE)" being the previously named "Neurodegeneration-Related" with *APOE* removed. MHC-I and MHC-II included all of the annotated genes of the MHC-I or MHC-II loci along with *B2M* (MHC-I) and *CD74* (MHC-II). Natural Killer Cell markers were hand-selected using tools on Immgen website (http://www.immgen.org). Ferritin+ cluster gene set are those genes elevated in the Ferritin+ cluster in both syn18485175 and GSE133433 (blue genes in top right quadrant of panel (C) right plot). Complete gene lists can be found in Data S3 columns S and T.

Panel 3. Analysis of multiple sclerosis sn/scRNA-Seq datasets

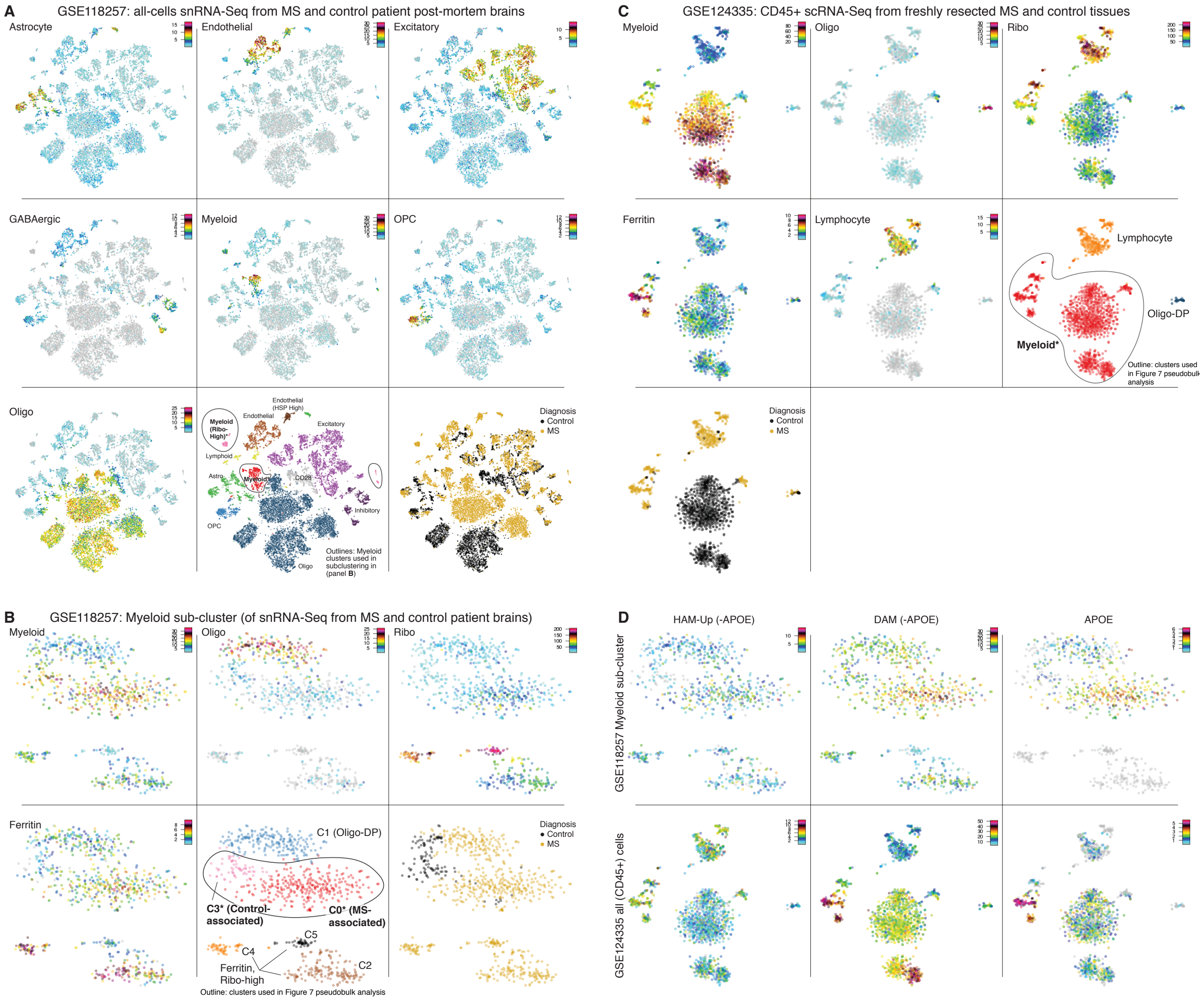

Clusters of myeloid cells from post-mortem and surgically resected MS tissue have elevated HAM and DAM signature expression relative to control myeloid cells.

(A) Previously described cell-type marker sets enable identification of myeloid and other cells in snRNA-Seq of post-mortem MS and control brains. tSNE plots were colored by indicated gene set scores (see Methods), clusters interpretation (center frame of bottom row) or subject diagnosis (bottom right frame). Two myeloid clusters (indicated with **Bold***) were taken into subclustering in (B).

(B) Myeloid clusters from (A) were subclustered and tSNE plots were colored with indicated gene sets, clusters, or diagnosis. Clusters C0 and C3 (indicated with **Bold***) were taken into pseudobulk analysis (main text Figure 7).

(C) Like (A), but for a dataset of fresh surgically resected CD45+ scRNA-Seq (GSE124335). Gene sets not shown had very low expression, as expected for CD45+ cells. Only Myeloid cluster (labeled with **Bold***) was taken into pseudobulk analysis (main text Figure 7).

(D) Gene set analysis of snRNA-Seq myeloid subcluster (top row) or all cells from CD45+ scRNA-Seq (bottom row) with HAM-Up and DAM (without *APOE*) gene sets (first two columns) as well as *APOE* gene expression (last column). HAM-Up, DAM, and *APOE* appear to be elevated to some extent in MS cells. Complete gene lists for all panels can be found in Data S3 columns P, S, and T.
